# Ubiquitin ligase TRIM71 suppresses ovarian tumorigenesis by degrading mutant p53

**DOI:** 10.1038/s41419-019-1977-3

**Published:** 2019-09-30

**Authors:** Yajie Chen, Qian Hao, Jieqiong Wang, Jiajia Li, Canhua Huang, Yu Zhang, Xiaohua Wu, Hua Lu, Xiang Zhou

**Affiliations:** 1 Cancer Institute, Fudan University Shanghai Cancer Center, Fudan University, Shanghai, 200032 China; 2 Department of Radiation Oncology, Fudan University Shanghai Cancer Center, Fudan University, Shanghai, 200032 China; 3 Department of Oncology, Shanghai Medical College, Fudan University, Shanghai, 200032 China; 4 Department of Gynecological Oncology, Fudan University Shanghai Cancer Center, Fudan University, Shanghai, 200032 China; 5 Department of Obstetrics and Gynecology, Xiangya Hospital, Central South University, Changsha, 410008 China; 6 Hunan Provincial Gynecological Cancer Diagnosis and Treatment Engineering Research Center, Changsha, 410008 China; 7 Department of Biochemistry & Molecular Biology and Tulane Cancer Center, Tulane University School of Medicine, New Orleans, LA 70112 USA; 8 Institutes of Biomedical Sciences, and Key Laboratory of Medical Epigenetics and Metabolism, Fudan University, Shanghai, 200032 China

**Keywords:** Tumour biomarkers, Tumour-suppressor proteins

## Abstract

Hotspot p53 mutants augment cancer cell proliferation, metastasis and metabolism through their gain-of-function (GOF). Ovarian cancer sustains the highest frequency of *TP53* mutations, but the mechanisms underlying regulation of mutant p53s’ GOF in this type of cancer remain incompletely understood. Herein, we identified the E3-ubiquitin ligase TRIM71 as a novel mutant p53-binding protein. Ectopic TRIM71-induced ubiquitination and proteasomal degradation of mutant p53 by binding to its transactivation (TA) domain, and inhibited the expression of a broad spectrum of mutant p53 target genes. Ectopic TRIM71 also restrained, whereas ablation of TRIM71 endorsed, ovarian carcinoma cell growth in vitro and in vivo. Significantly, TRIM71 overexpression is highly associated with favorable prognosis, particularly, in *TP53*-mutated ovarian carcinomas. Altogether, our findings unveil the anti-tumor function of TRIM71 in ovarian cancer development and prognosis by downregulating mutant p53s.

## Introduction

The tumor suppressor p53 prevents malignancies, but its activity is prohibited almost in all types of human cancers via various mechanisms^1^. One of the efficient ways to inactivate p53 is to mutate the *TP53* gene^2–4^. Cancer-associated mutations of *TP53* not only abolish the tumor suppressive function of wild-type p53 (wtp53), leading to “loss of function”, but also cause “dominant-negative” effect by repressing the remaining wild-type allele. In addition, a number of missense mutant p53s (mtp53s), particularly the “hotspot” mutants, have been found to acquire gain-of-function (GOF) to endorse tumor growth, metastasis, and drug resistance^2–4^. Most mtp53s lose the ability to directly bind to and regulate the p53-responsive DNA elements. Instead, they indirectly induce or repress gene expression through interaction with other transcription factors or co-factors^2–4^. Recently, we have demonstrated that CDK4/Cyclin D1-phosphorylated p53-R249S interacts with and stabilizes c-MYC, resulting in activation of ribosomal gene transcription and hepatocellular carcinoma cell growth^5^. In clinic, the frequency of *TP53* mutations is up to 96–99% in the high-grade serous ovarian cancer^6,7^ that is the most common and aggressive histotype of ovarian cancer. Several studies have inferred that mtp53s could be a biomarker or pivotal to the development of ovarian cancer^8,9^. Also, a few compounds, such as APR-246 and AZD1775, have been attested to be beneficial to advanced and drug-refractory ovarian cancer by targeting mtp53-associated pathways^10–12^. However, it still remains unclear if and how these mtp53s are regulated in ovarian cancer, and if potential mtp53 regulators play a role in the progression of the mtp53-driven cancers.

In our attempt to address these questions, we initiated a screen to explore mtp53-interacting proteins in primary human ovarian cancer tissues, and identified an E3-ubiquitin ligase, named TRIM71 (also known as LIN41), as a novel regulator of mtp53s. TRIM71 was reported to be involved in embryonic development, neurogenesis, and stem cell renewal by degrading its binding mRNAs or inhibiting mRNA translation through various mechanisms^13–15^. Yet, its role in cancer development has remained elusive. Our further characterization of the TRIM71–mtp53 functional interactions revealed that this E3-ubiquitin ligase has a tumor-suppressive role by promoting ubiquitination-dependent proteolysis of mtp53s and thus repressing the expression of mtp53 target genes. Consistently, ectopic TRIM71 suppressed ovarian cancer cell proliferation in vitro and ovarian tumor growth in vivo, whereas RNAi- or CRISPR-Cas9-mediated ablation of endogenous TRIM71 promoted ovarian cancer cell growth and migration in vitro and/or in vivo. In line with these results, the level of TRIM71 is inversely correlated with the expression of mtp53 and its target genes in ovarian carcinomas, and is positively associated with improved patient survival. Hence, our studies as detailed below unravel the tumor-suppressive function of TRIM71 in ovarian cancer through inhibition of mtp53s.

## Materials and methods

### Cell culture and transient transfection

Human cancer cell lines ES-2, OVCA420, OVCAR433, TOV112D, HT-29, HCT116^p53−/−^ and mouse MEFs^p53−/−^;^Mdm2−/−^ were cultured in Dulbecco’s modified Eagle’s medium supplemented with 10% fetal bovine serum, 100 U/ml penicillin and 100 µg/ml streptomycin. All cells were maintained at 37 °C in a 5% CO_2_-humidified atmosphere. Cells were seeded on the plate the day before transfection, and then transfected with plasmids or siRNAs as indicated in the figure legends using Hieff Trans liposomal transfection reagent following the manufacturer’s protocol (Yeasen, Shanghai, China). Cells were harvested at 30–48 h post transfection for future experiments. Cycloheximide and the proteasome inhibitor MG132 were purchased from Sigma-Aldrich (St. Louis, MO, USA). The HSP90 inhibitor Tanespimycin (17-AAG) and the Mdm2 antagonist Nutlin-3 were purchased from Selleck (Houston, TX, USA).

### Plasmids and antibodies

The Flag-tagged TRIM71-expressing plasmid was purchased from Vigene Biosciences, Inc. (Shandong, China). The Myc-tagged TRIM71 plasmid was generated by inserting the full-length cDNA amplified by PCR into the pcDNA3.1/Myc-His vector between the *Xba*I and *Not*I sites, using the following primers, 5′-GCTCTAGAATGGCTTCGTTCCCCG-3′ and 5′-ATAAGAATGCGGCCGCGAAGACGAGGATTCGATTGT-3′. The Myc-tagged plasmids expressing TRIM71 fragment, aa 1–577, 97-868, or 577-868, were generated by the same approach using the corresponding primers. The plasmids expressing HA-MDM2 and wtp53 were described previously^16,17^. The plasmids encoding non-tagged mtp53 (R175H, Y220C, S241F, R248W, and R273H) and Flag-tagged mtp53 fragments (S241F and R273H) were generated using the KOD-plus-mutagenesis kit (Toyobo, Japan). The plasmids encoding His-tagged point-mutation of ubiquitins were described previously^18^. The anti-Flag (Catalog No. F1804, Sigma-Aldrich), anti-Myc (Catalog No. 60003-1, Proteintech, Hubei, China), anti-GFP (Catalog No. 66002-1, Proteintech), anti-TRIM71 (E-1, Catalog No. sc-393352, Santa Cruz Biotechnology, Santa Cruz, CA, USA), anti-p53 (DO-1, Catalog No. sc-126, Santa Cruz Biotechnology), anti-p21 (Catalog No. #2947, Cell Signaling Technology, Danvers, MA, USA), anti-PUMA (Catalog No. #12450, Cell Signaling Technology), anti-MDM2 (Catalog No. ab16895, Abcam, Cambridge, MA, USA), anti-GAPDH (Catalog No. 60004-1, Proteintech), and the secondary antibodies for rabbit (Catalog No. ARG65351, Arigo) and mouse (Catalog No. ARG65350, Arigo), and the light chain-specific secondary mouse antibody (Catalog No. 115-035-174, Jackson) were commercially purchased.

### Reverse transcription and quantitative RT-PCR analyses

Total RNA was isolated from cells using RNAiso Plus (Takara, Japan) following the manufacturer’s protocol. Total RNAs of 0.5–1 μg were used as templates for reverse transcription using PrimeScript RT reagent Kit with gDNA Eraser (Takara, Japan). Quantitative RT-PCR (RT-qPCR) was conducted using TB Green Premix according to the manufacturer’s protocol (Takara, Japan). The primers for TRIM71, p53, CXCL1, MAP2K3, NFKB2, MMP3, MMP13, ITGA6, c-MYC, RANGAP1, ARHGDIA, TDP2, and GAPDH cDNA detection are as follows: TRIM71, 5′-CCTGGAGGAACGCGAGTGTGA-3′ and 5′-GGGCCAGTAGGATGTCTAGC-3′; p53, 5′-CCCAAGCAATGGATGATTTGA-3′ and 5′-GGCATTCTGGGAGCTTCATCT-3′; CXCL1, 5′-CTGAACAGTGACAAATCCAAC-3′ and 5′-CCTAAGCGATGCTCAAACAC-3′; MAP2K3, 5′-CTGCGGTTCCCTTACGAGT-3′ and 5′-GCAATGTCCGTCTTCTTGGT-3′; NFKB2, 5′-GGGGCATCAAACCTGAAGATTTCT-3′ and 5′-TCCGGAACACAATGGCATACTGT-3′; MMP3, 5′-ATCCTACTGTTGCTGTGCGTG-3′ and 5′-ACTTCTGCATTTCTCGGATTT-3′; MMP13, 5′-GAATTAAGGAGCATGGCGACT-3′ and 5′-CTAAGGAGTGGCCGAACT-3′; ITGA6, 5′-GCACGCGGATCGAGTT-3′ and 5′-CTCGGGATTCCTGCTTCGTAT-3′; c-MYC, 5′-GGAGATCCGGAGCGAATAG-3′ and 5′-CCTTGCTCGGGTGTTGTAAGT-3′; RANGAP1, 5′-GCTCCAAGGGTGCAGTTG-3′ and 5′-GCAGCATCCCTCTTGATTTC-3′; ARHGDIA, 5′-AGCCTGCGAAAGTACAAGGA-3′ and 5′-GGTCAGGCCAGTCACCAC-3′; TDP2, 5′-ATGCTGCGGAACGAATGAAT-3′ and 5′-CCACCACATCTGGTAACCTCTC-3′; GAPDH, 5′-GGAGCGAGATCCCTCCAAAAT-3′ and 5′-GGCTGTTGTCATACTTCTCATGG-3′.

### Immunoblotting

Cells were harvested and lysed in lysis buffer consisting of 50 mm Tris/HCl (pH7.5), 0.5% Nonidet P-40 (NP-40), 1 mm ethylenediaminetetraacetic acid, 150 mm NaCl, 1 mm dithiothreitol, 0.2 mm phenylmethylsulfonyl fluoride, 10 mm pepstatin A and 1 mm leupeptin. Equal amounts of clear cell lysates (20–80 μg) were used for immunoblotting (IB) analyses as described previously^16^.

### Immunoprecipitation

Immunoprecipitation (IP) assays were conducted using antibodies as indicated in the figure legends. In brief, ~ 500–1000 μg of proteins were incubated with the indicated antibody at 4 °C for 4 h or overnight. Protein A or G beads (Santa Cruz Biotechnology) were then added, and the mixture was incubated at 4 °C for additional 1–2 h. Beads were washed at least three times with lysis buffer. Bound proteins and 10% inputs were detected by IB with antibodies as indicated in the figure legends.

### In vivo ubiquitination assay

HCT116^p53−/−^ cells were transfected with combinations of plasmids encoding mtp53 (S241F or R273H), HA-MDM2, His-Ub or Flag-TRIM71 as indicated in the figure legends. At 48 h after transfection, cells were harvested and split into two aliquots, one for IB and the other for the ubiquitination assay. In brief, cell pellets were lysed in buffer I (8 m urea, 0.1 m Na_2_HPO4/NaH_2_PO4 (pH 8.0), 10 mm Tris-HCl (pH 8.0), 10 mm β-mercaptoethanol, 5 mm Imidazole) and incubated with Ni-NTA beads (Takara, Japan) that capture His-tagged proteins/complex at room temperature for 4–6 h. Beads were washed twice with buffer I, then buffer II (8 m urea, 0.1 m Na_2_HPO4/NaH_2_PO4 (pH 6.3), 10 mm Tris-HCl (pH 6.3), 10 mm β-mercaptoethanol). The captured proteins were eluted and analyzed by IB with the indicated antibodies.

### RNA interference and generation of lentiviral particles

The siRNA against TRIM71 and p53 were synthesized and purified by GenePharma (Shanghai, China). The siRNA sequences against human TRIM71 or p53 are 5′-GTGCATAACAGTAACTAGA-3′ (si-TRIM71-1), 5′-GATTCTACGATTGCTCTGT-3′ (si-TRIM71-2), and 5′-GUAAUCUACUGGGACGGAAtt-3′ (si-p53)^17^. The siRNAs (50–100 nm) were introduced into cells using Hieff Trans liposomal transfection reagent following the manufacturer’s protocol (Yeasen). Cells were harvested 48–72 h after transfection for IB or RT-qPCR. The pCDH-TRIM71 plasmid was generated by inserting the full-length cDNA amplified by PCR into the lentivirus-based pCDH vector at *Xba*I and *Not*I sites using the primers described above. The HEK293T cells were transfected with pCDH vector or pCDH-TRIM71, along with the packaging plasmids, psPAX2 and pMD2.G. The virus particles were collected 48 h after transfection and then used for cell infection.

### CRISPR/Cas9-mediated gene editing

The CRISPR/Cas9 targeting vector lentiCRISPR v2 was purchased from Addgene (Cambridge, MA, USA). The sgRNA for TRIM71 was designed at http://crispr.mit.edu/, and the sequence of the high-scored sgRNA was 5′-CTCGCAGACGTCCACGTCGT-3′. For sgRNA subcloning, the lentiCRISPR v2 vector was digested with BsmBI and ligated with BsmBI compatible annealed oligoes. The lentiviruses were generated as described above. The infected ES-2 cells were selected by 1 μg/ml puromycin for > 7 days.

### Cell viability assay

To assess the long-term cell survival, the Cell Counting Kit-8 (CCK-8) (Dojindo, Japan) was used according to the manufacturer’s instructions. Cell suspensions were seeded at 2000 cells per well in 96-well culture plates at 12 h post transfection. Cell viability was determined by adding WST-8 at a final concentration of 10% to each well, and the absorbance of the samples was measured at 450 nm using a Microplate Reader every 24 h for 5 days. The cell proliferation curves were plotted using the absorbance at each time point.

### Colony formation assay

Cells were re-suspended into single-cell suspensions and plated into six-well plates 24 h post transfection. The medium was changed every 3 days until the colonies were visible. After 2 weeks, the cells were fixed by methanol and stained with 0.1% crystal violet at RT for 30 min. The colonies were manually counted.

### Cell wound-healing and invasion assays

For the wound-healing assay, cells were seeded into six-well plates and allowed to grow to 90–95% confluence. The cells were washed with PBS to remove cell debris, cultured with serum-free medium, and monitored. Images were captured by phase-contrast microscopy at 0, 12, 24, and 36 h after wounding. Invasion assays were performed using Transwell chamber inserts in a 24-well plate. In total, 5 × 10^4^ cells suspended in 100 µL of serum-free medium were added to the upper chamber. The lower chambers were filled with the normal culture medium. The cells were incubated for 12–24 h at 37 °C. After incubation, the cells on the upper surface were scraped and washed away, and the cells on the lower surface were fixed with methanol and stained with 0.1% crystal violet. The number of invaded cells was counted in at least five randomly selected fields under an optical microscope by image J software.

### Mouse xenograft experiments

Female BALB/c nude mice, 4–5 weeks old, were purchased from the Department of Laboratory Animal Science in Shanghai Medical College of Fudan University. To evaluate the effect of TRIM71 overexpression on tumor growth in vivo, mice were subcutaneously inoculated with 5 × 10^5^ ES-2 cells stably expressing pCDH vector or pCDH-TRIM71 in the left and right flanks, respectively. Inversely, to determine the effect of TRIM71 depletion on tumor growth, mice were bilaterally inoculated with 5 × 10^5^ ES-2 cells stably expressing Ctrl-Cas9 or TRIM71-Cas9 per flank. Tumor growth was monitored every other day with electronic digital calipers in two dimensions. Tumor volume was calculated according to the formula: volume = length × width^2^ × 0.5. Tumors were then harvested, weighed, and subjected to IHC and IB analyses.

### Database of cancer patients

From Cancer Genomics Database (http://www.cbioportal.org/), we downloaded three digital gene expression files of 307 ovarian cancer tumors, 373 liver cancer tumors, and 498 prostate cancer tumors, which were generated using a RNA-seq platform by the Cancer Genome Atlas (TCGA)^19,20^. The mRNA expression correlations between TRIM71 and other genes were evaluated using Pearson correlation coefficient. The significant differences of survival of the patients were analyzed by the Kaplan–Meier statistics. The KM plotter database (www.kmplot.com) was also used for survival analysis of cancer patients^21^. The Oncomine database (https://www.oncomine.org) was used to analyze the expression of TRIM71 in cancers vs. normal tissues.

### Statistics

All in vitro experiments were performed in biological triplicate. The Student’s *t* test or one way analysis of variance was performed to evaluate the differences between two groups or more than two groups. The Kaplan–Meier statistics were used to analyze the significant difference of patient survival. The Cox univariate proportional hazards regression models was used to determine the independent clinical factors based on the investigated variables. Pearson’s correlation was performed to analyze the correlation of the gene expression profiling. *p* < 0.05 was considered statistically significant. All the data are presented as mean ± SD.

## Results

### Identification of TRIM71 as a mutant p53-binding protein in ovarian cancer

**Fig. 1:**
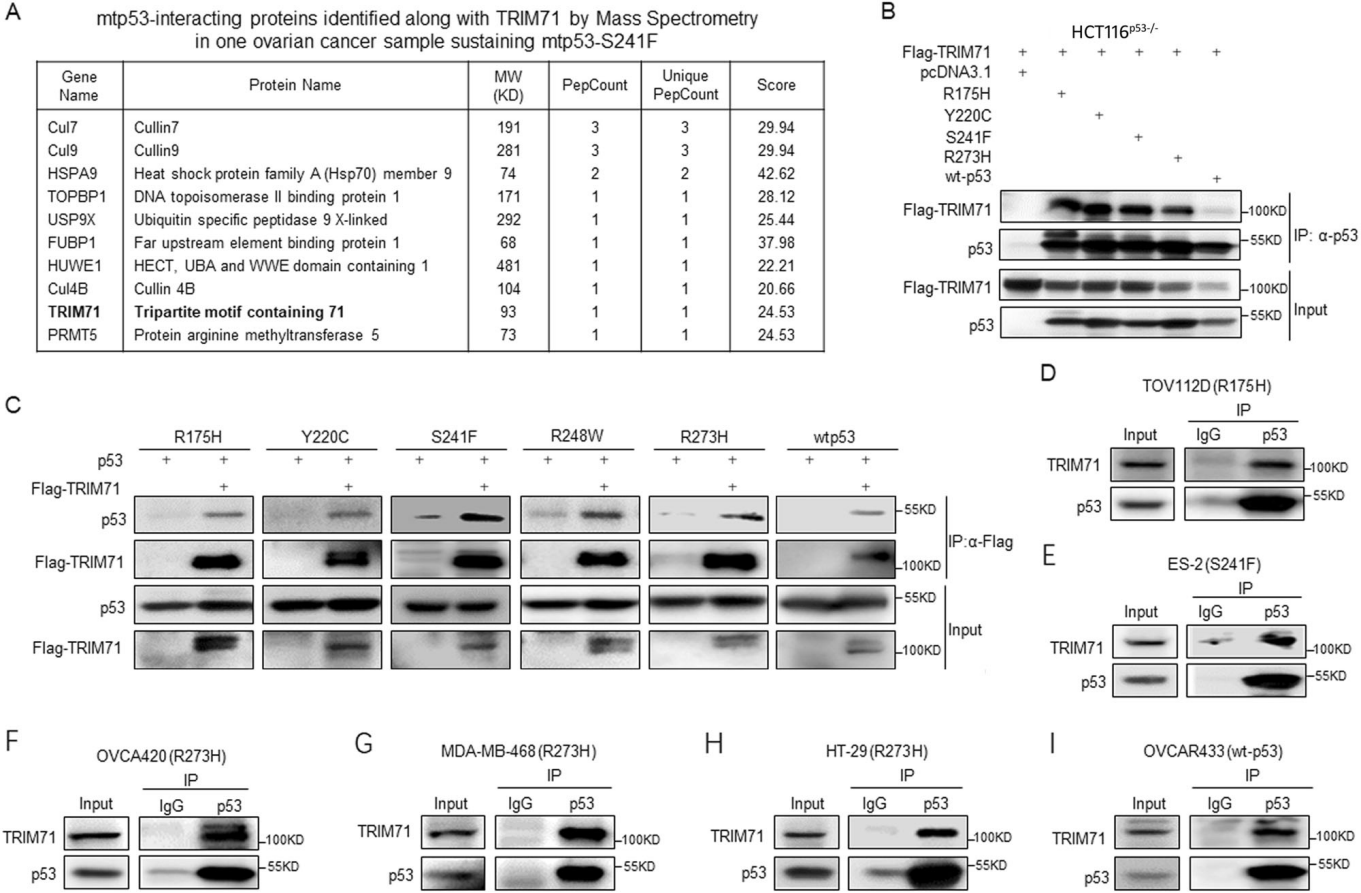
TRIM71 is a novel mutant p53-binding protein in ovarian cancer. **a** A list of mtp53-interacting proteins, including TRIM71, identified in the ovarian cancer tissue sustaining mtp53-S241F. All the proteins other than TRIM71 are known wtp53- or mtp53-interacting proteins. **b**, **c** Exogenous TRIM71 preferentially binds to exogenous mtp53s (R175H, Y220C, S241F, R248W, and R273H) by reciprocal IP assays. HCT116^p53−/−^ cells were transfected with plasmids encoding Flag-TRIM71 and mtp53s or wtp53 followed by co-IP-IB assays using antibodies as indicated. **d**–**i** Endogenous interactions between TRIM71 and p53s in the ovarian cancer cell lines, TOV112D (R175H), ES-2(S241F), OVCA420 (R273H), MDA-MB-468 (R273H), HT-29 (R273H), and OVCAR433(wtp53), as determined by co-IP-IB assays using anti-p53 antibody (DO-1)

**Fig. 2:**
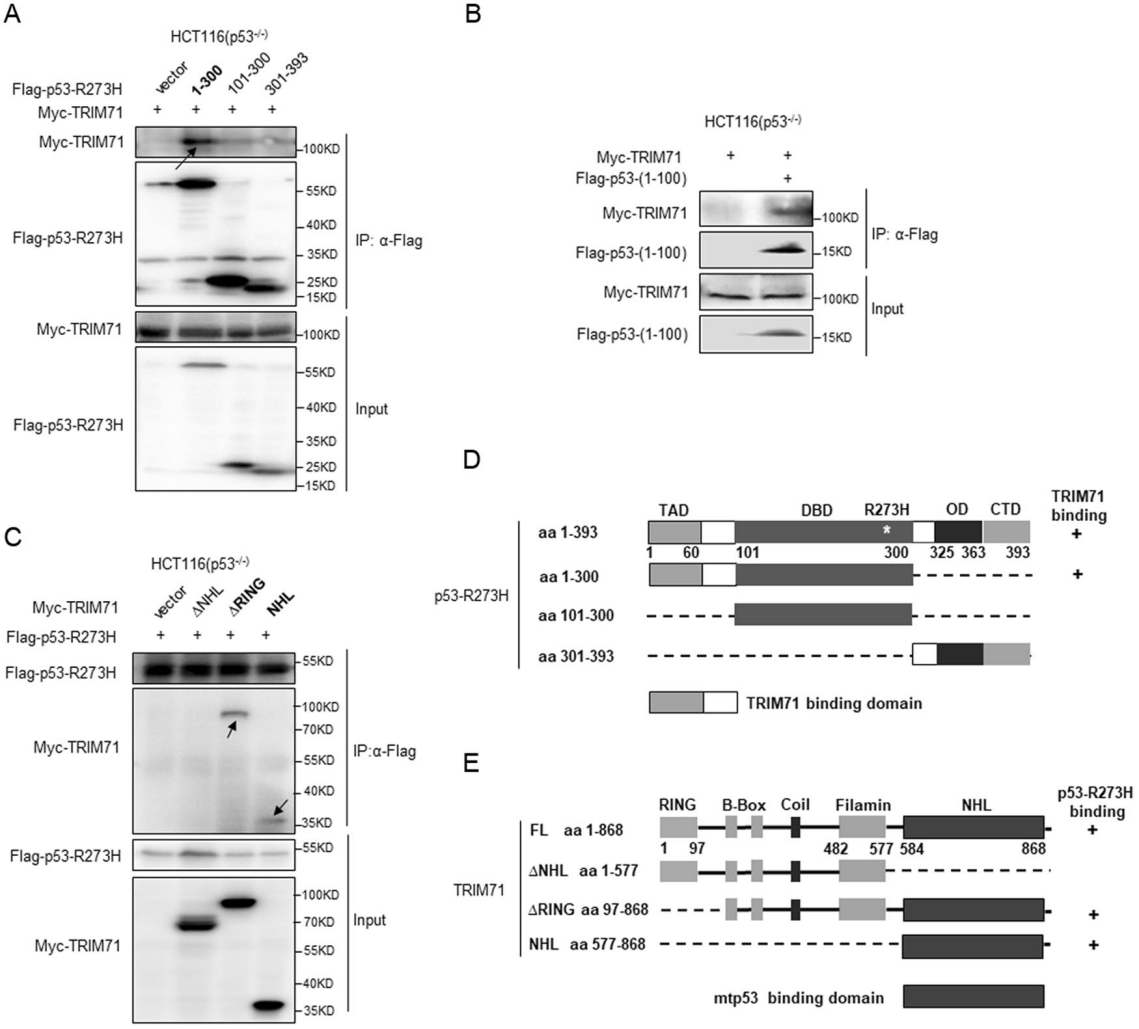
The TRIM71-NHL domain interacts with the TA domain of mutant p53-R273H. **a** Mapping the TRIM71 binding domain of mtp53-R273H. HCT116^p53−/−^ cells were transfected with the plasmid encoding Flag-p53-R273H fragment, aa 1–300, aa 101–300 or aa 301–393, along with the Myc-TRIM71-encoded plasmid. Co-IP assays were performed using the anti-Flag antibody followed by IB with the anti-Myc antibody. **b** HCT116^p53−/−^ cells were transfected with plasmids encoding Myc-TRIM71 and Flag-p53-(aa 1–100) followed by co-IP-IB assays using antibodies as indicated. **c** Mapping the mtp53-R273H binding domain of TRIM71. HCT116^p53−/−^ cells were transfected with the plasmids encoding each Myc-TRIM71 fragment along with the Flag-p53-R273H-expressing plasmid. Co-IP-IB assays were performed using antibodies as indicated. **d** A schematic diagram of TRIM71-binding region on mtp53-R273H. TAD, Transactivation domain (1–42); DBD, DNA-binding domain (101–306); OD, Oligomerization domain (307–355); CTD: C-terminal regulatory domain. **e** A schematic diagram of mtp53-R273H binding region on TRIM71

To decipher the molecular basis for the development of ovarian malignancy by looking for mtp53-binding proteins, we conducted co-IP with the anti-p53 antibody coupled with mass spectrometry (MS) analysis using a primary ovarian tumor sample with p53-S241F as detected by sequencing the *TP53* gene exons. This resulted in identification of TRIM71 as one of the p53-S241F-binding proteins (Fig. 1a). Also, there were several known wtp53- or mtp53-interacting proteins, such as Cullins^22^, HSPA9^23^, TOPBP1,^24^ and HUWE1^25^, found in the same tumor sample, indicating the reliability of this primary screening (Fig. 1a). To validate this interaction, we performed a set of co-IP assays and found that ectopic TRIM71 indeed bound to all the mtp53s tested, including R175H, Y220C, and R273H, in addition to S241F (Fig. 1b). The reverse co-IP assays using the anti-Flag antibody again confirmed the interactions of TRIM71 with mtp53s, such as R175H, Y220C, S241F, R248W, and R273H (Fig. 1c). However, to a much less degree, a small amount of TRIM71-wtp53 complexes were detected (Fig. 1b, c), which is consistent with a recent report showing the regulation of wtp53 by TRIM71 during early brain development^26^. We also verified the endogenous TRIM71–mtp53 interactions in various cancer cell lines, including the ovarian cancer cell lines, ES-2 (p53-S241F), TOV112D (p53-R175H), OVCA420 (p53-R273H), and OVCA433 (wtp53), the breast cancer cell line MDA-MB-468 (p53-R273H), and the colon cancer cell line HT-29 (p53-R273H) (Fig. 1d–i). Binding domain mapping revealed that TRIM71 binds to the N-terminal transactivation (TA) domain (amino acid (aa) 1–100), but not the aa 101–300 or aa 301–393 of mtp53 (Fig. 2a, b, d), and that mtp53 strongly binds to the TRIM71-NHL domain, as its depletion (ΔNHL) completely abolishes the TRIM71–mtp53 interaction (Fig. 2c, e). Together, these results indicate that TRIM71 binds to the TA domain of mtp53s via its NHL domain in cancer cells.

### TRIM71 induces ubiquitination and proteasomal degradation of mutant p53s

**Fig. 3:**
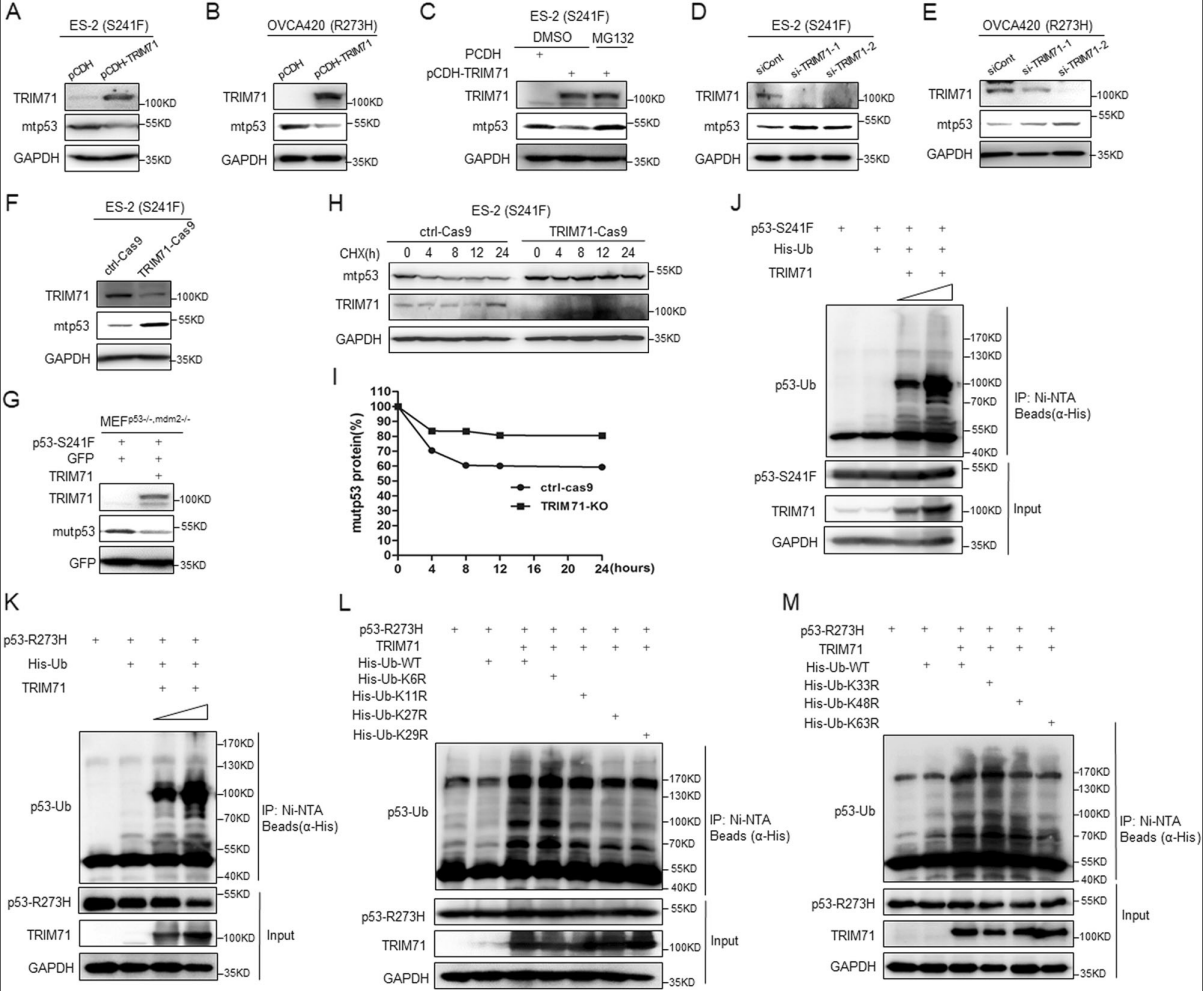
TRIM71 promotes ubiquitination and proteasomal degradation of mutant p53s. **a**, **b** Ectopic expression of TRIM71 reduces the mtp53 protein levels in ES-2 and OVCA420 cells. **c** The proteasome inhibitor MG132 blocks TRIM71-mediated mtp53 degradation in ES-2 cells. MG132 (20 μm) was supplemented for 6 h prior to cell harvest. **d**, **e** Knockdown of TRIM71 increases mtp53 protein levels in ES-2 and OVCA420 cells. **f** CRISPR-Cas9-mediated ablation of TRIM71 elevates p53-S241F protein expression in ES-2 cells. **g** Overexpression of TRIM71 induces degradation of exogenous mtp53 in the p53/Mdm2 double-knockout MEFs. Combinations of plasmids encoding Myc-TRIM71, p53-S241F, or EGFP as the control were introduced into the MEFs, followed by the IB assay using antibodies as indicated. **h**, **i** The mtp53’s half-life is extended upon TRIM71 depletion. The Ctrl-Cas9 and TRIM71-Cas9 cell lines were treated with 100 mg/ml of CHX and harvested at different time points as indicated. The mtp53 protein was detected by the IB assay **h**, and quantification of p53/GAPDH ratio is shown in the panel **I**. **j**, **k** TRIM71 promotes ubiquitination of mtp53, S241F and R273H. HCT116^p53−/−^ cells were transfected with combinations of plasmids encoding mtp53s (S241F or R273H), Flag-TRIM71 or His-Ub as indicated, and treated with MG132 (20 μm) for 6 h before harvested for in vivo ubiquitination assay. **l**, **m** TRIM71 mediates p53-R273H ubiquitination through K11-, K27-, K29-, and K63-linked ubiquitin chains. HCT116^p53−/−^ cells were transfected with combinations of plasmids encoding p53-R273H, Flag-TRIM71, and His-Ub or His-Ub-mutants, and treated with MG132 (20 μm) for 6 h before harvested for in vivo ubiquitination assay. Bound and input proteins were detected by IB assays with the indicated antibodies

As TRIM71 is an E3-ubiquitin ligase with a functional RING domain, we tested if TRIM71 regulates mtp53 protein turnover. First, we introduced TRIM71 into ES-2 and OVCA420 cells with a lentivirus-based vector, and found that ectopic TRIM71 diminishes the protein levels of p53-S241F and R273H (Fig. 3a, b), whereas this reduction is completely restored by supplementation of the proteasome inhibitor MG132 (Fig. 3c). Conversely, ablation of TRIM71 by two independent siRNAs (Fig. 3d, e) or by the CRISPR-Cas9 system (Fig. 3f) led to marked elevation of mtp53 expression. As MDM2 is the central regulator of both wt and mtp53^27,28^, we tested if TRIM71-mediated mtp53 degradation is dependent on MDM2 or not using p53/Mdm2 double-knockout MEFs. As illustrated in Fig. 3g, TRIM71 overexpression could reduce the level of exogenous mtp53 in the absence of MDM2. Consistently, depletion of TRIM71 prolonged the half-life of mtp53 protein as indicated by the cycloheximide-chase experiment (Fig. 3h, i). Then, we performed in vivo ubiquitination assays to show that ectopic expression of TRIM71 displays a dose-dependent effect in stimulating ubiquitination of mtp53s, S241F and R273H (Fig. 3j, k). Next, we sought to determine which lysine(s) of ubiquitin might be required for TRIM71-induced mtp53 ubiquitination. To this end, we conducted a set of in vivo ubiquitination assays using plasmids encoding wild-type or lysine-mutant ubiquitins, and found that the lysines K11, K27, K29, and K63 are responsible for mtp53 ubiquitination (Fig. 3l, m). As the K11, K29, and K63-linked ubiquitin chains have all been reported to trigger proteasomal degradation^29^, we believe that they are critical for mtp53 degradation mediated by TRIM71. Altogether, these results demonstrate that the E3-ubiquitin ligase TRIM71 undermines mtp53 protein stability by inducing its ubiquitination and proteasomal degradation.

### Inhibition of mutant p53 target gene expression by TRIM71

**Fig. 4:**
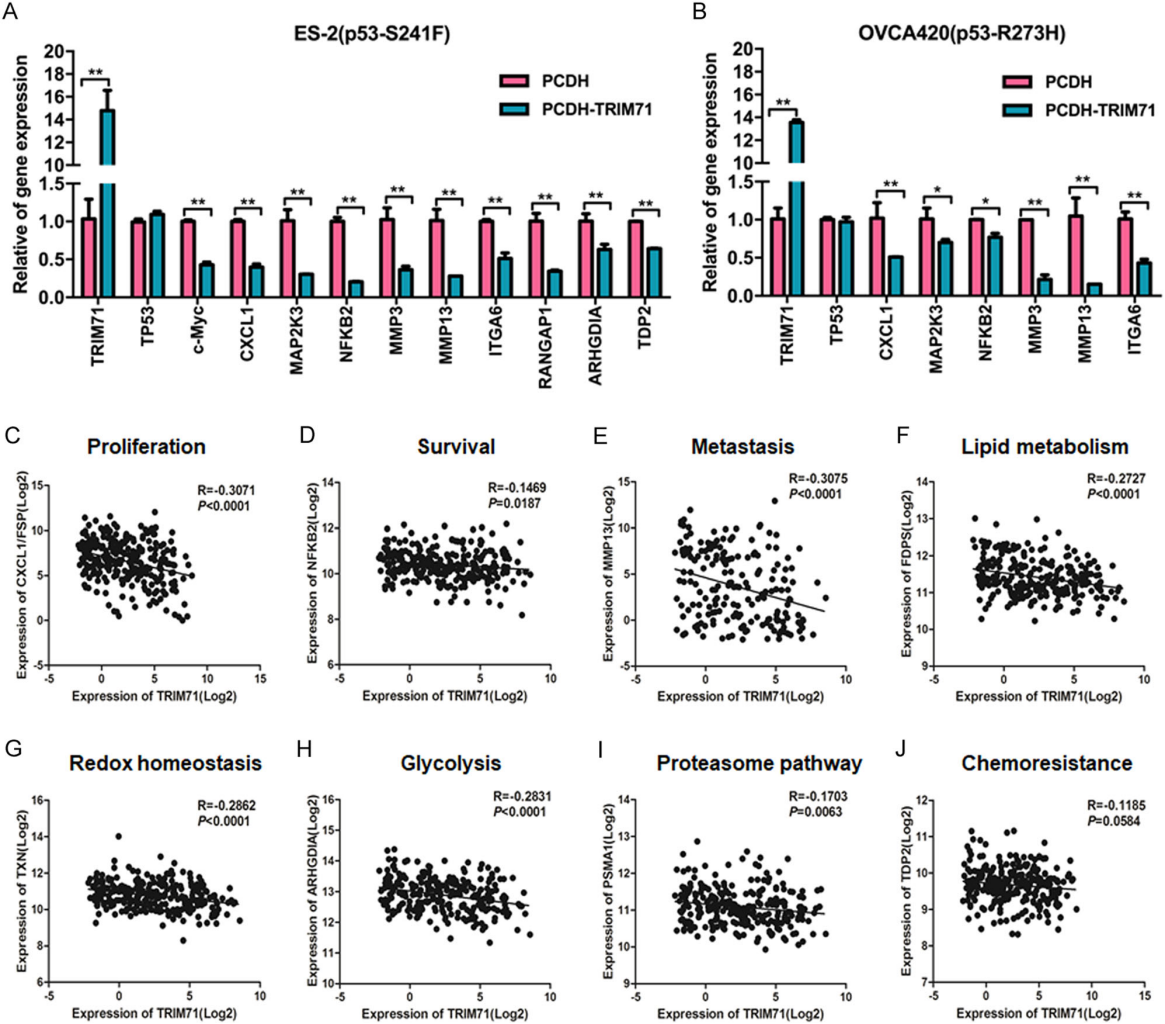
TRIM71 inhibits mutant p53 target gene expression in ovarian cancer. **a**, **b** The expression of mtp53s and their target genes was assessed by RT-qPCR upon ectopic expression of TRIM71 in ES-2 and OVCA420 cells. Data are represented as mean ± SD, *n* = 3. *p < 0.05, **p < 0.01 by two tailed *t* test. **c**–**j** The inverse correlation between expression of TRIM71 and mtp53 target genes involved in a wide range of biological outcomes, such as proliferation **c**, survival **d**, metastasis **e**, metabolism **f**, redox homeostasis **g**, glycolysis **h**, proteasome pathway **i**, and chemoresistance **j** in ovarian cancer

Although mtp53s usually lack the ability to associate with the p53-responsive DNA elements, they can still regulate gene expression indirectly by binding to other transcription factors or co-factors^2–4^. We therefore examined if TRIM71 suppresses the transcriptional function of mtp53s by reducing their protein levels. As expected, stable overexpression of TRIM71 did not affect mRNA expression of mtp53s in ES-2 and OVCA420 cell lines, but globally prohibited the expression of their target genes involved in different GOFs, such as c-MYC, CXCL1, MAP2K3, and NFKB2 required for cancer cell growth and survival, MMP3, MMP13, and ITGA6 for EMT and metastasis, RANGAP1 and ARHGDIA for GTP metabolism and glycolysis, and TDP2 for chemoresistance (Fig. 4a, b).

Next, we explored if TRIM71-mediated inactivation of mtp53 might be reflected in primary ovarian cancers. Concordantly, by mining the TCGA database, we unveiled a negative correlation between the expression of TRIM71 and a broad spectrum of mtp53 target genes (Fig. 4c–j and S1), further suggesting that TRIM71 might downregulate mtp53 target genes in ovarian carcinomas. These correlations also suggest that TRIM71 might regulate ovarian cancer cell proliferation (Fig. 4c and S1a–d), survival (Fig. 4d and S1e–g), metastasis (Fig. 4e and S1h), lipid metabolism (Fig. 4f and S1i–o), redox homeostasis (Fig. 4g and S1t–y), glycolysis (Fig. 4h), proteasome pathway (Fig. 4i and S1p–s), and chemoresistance (Fig. 4j) by antagonizing mtp53s’ GOFs. We then inquired if these negative correlations in patients are dependent on mtp53. Thus, we performed the same analysis in some prostate cancers that sustain less frequency (~ 10–18%) of *TP53* mutations. Interestingly, none of the mtp53 target genes showed negative correlation with the TRIM71 expression (Fig. S2 and S3), indicating that TRIM71-mediated downregulation of these genes is mtp53-dependent. Finally, we showed an inverse correlation of TRIM71 with mtp53 protein expression in 11 ovarian cancer samples harboring *TP53* missense mutations (Fig. S4a) determined by *TP53*-exon sequencing (Fig. S4b, c). Taken together, these results demonstrate that TRIM71 suppresses the expression of mtp53 target genes in ovarian cancer and suggest that this might lead to impaired mtp53s’ GOF, which is addressed as follows.

### TRIM71 suppresses ovarian cancer cell growth and invasion through inactivation of mutant p53s

The GOF of the hotspot mutant, p53-R273H, has been well-documented, whereas there is little knowledge about the activity of p53-S241F. Thus, we wanted to determine the function of p53-S241F, with R273H as a control, in ovarian cancer cells. Knockdown of each of the two mtp53s did not affect the expression of the p53 target gene p21 in ES-2 or OVCA420 cells (Fig. S5a, b). Likewise, the DNA damage-triggering agents, 5-fluorouracil and Cisplatin, failed to induce the expression of p53-S241F or the p53 target genes, MDM2, p21, and PUMA, in ES-2 cells (Fig. S5c). Of note, levels of p21 and PUMA moderately reduced upon DNA damage, suggesting that other mechanisms might be involved^30,31^. These data indicate that p53-S241F, like R273H, is deprived of the wild-type activity. Next, we conducted the cell viability and transwell assays, and found that both mutants are required for proliferation and invasion of ovarian cancer cells (Fig. S5d–f). Thus, these results confirm that both mtp53s possess oncogenic GOF, and the ES-2 and OVCA420 cell lines are suitable for further evaluation of the biological outcomes of the TRIM71–mtp53 cascade.

**Fig. 5:**
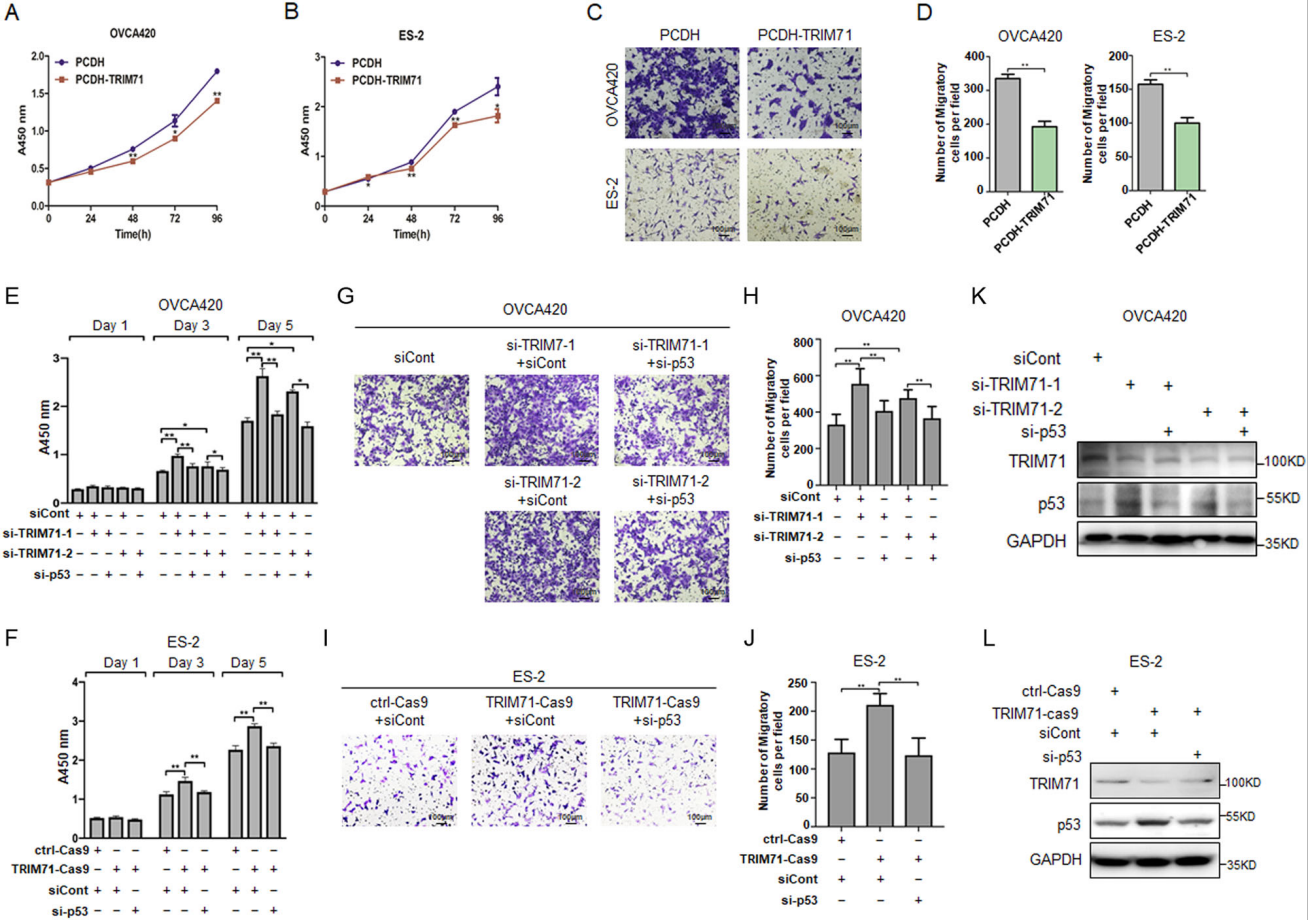
TRIM71 suppresses proliferation and invasion of ovarian cancer cells through inhibition of mutant p53s. **a**, **b** Lentivirus-based overexpression of TRIM71 inhibits proliferation of OVCA420 and ES-2 cells by the CCK-8 assays. **c**, **d** Lentivirus-based overexpression of TRIM71 inhibits invasion of OVCA420 and ES-2 cells by the transwell assays. The representative images and quantification analysis are shown in **c**, **d**, respectively. **e**, **f** Knockdown of mtp53 abrogates TRIM71 depletion-induced ovarian cancer cell proliferation. OVCA420 cells **e** and ES-2 cells stably expressing the ctrl-cas9 or TRIM71-cas9 **f** were transfected with indicated siRNAs followed by the CCK-8 assays. **g**–**j** Knockdown of mtp53 abrogates TRIM71 depletion-induced ovarian cancer cell invasion. OVCA420 cells **g**, **h** and ES-2 cells stably expressing the ctrl-cas9 or TRIM71-cas9 **i**, **j** were transfected with indicated siRNAs followed by transwell assays. The representative images and quantification analysis are shown in **g**, **i** and **h**, **j**, respectively. **k**, **l** Representative IB assays show the knockdown efficiency of TRIM71 and/or mtp53 in OVCA420 cells **k** and ES-2 cells **l**

As TRIM71 suppressed the expression of the survival- and metastasis-associated mtp53 target genes as described above (Fig. 4 and S1), we tested if this could be translated to its ability to suppress cell proliferation and invasion in OVCA420 and ES-2 cells. Indeed, stable overexpression of TRIM71 significantly impeded cell proliferation (Fig. 5a, b) and invasion (Fig. 5c, d) of the two cell lines. Conversely, knockdown of TRIM71 by two independent siRNAs markedly accelerated cell proliferation (Fig. S6a, b), enhanced the colony-forming ability (Fig. S6c, d), and promoted invasion of the two ovarian cancer cell lines (Fig. S6e, f). In line with these results, depletion of TRIM71 by the CRISPR-Cas9 system in ES-2 cells drastically boosted cell proliferation (Fig. S6g), migration (Fig. S6h, i), and invasion (Fig. S6j, k). To confirm that the effects of TRIM71 depletion are the consequence of stabilization of mtp53s, we simultaneously knocked down mtp53s to see if these phenotypes could be restored. As shown in Fig. 5e, f, ablation of p53-R273H or S241F abrogated TRIM71 depletion-induced cell proliferation. In addition, the transwell assays again validated that mtp53 is required for the enhanced cell invasion by depletion of TRIM71 (Fig. 5g–j). These effects were specific to deregulation of the TRIM71–mtp53 cascade, as efficiency of TRIM71 depletion and mtp53 restoration was validated (Fig. 5k, l). Collectively, the above results strongly demonstrate that TRIM71 can inhibit proliferation and metastasis of ovarian cancer cells that sustain GOF mutations of p53.

Interestingly, a recent study has described the crosstalk between TRIM71 and wtp53 in neurogenesis^26^, and we also found that TRIM71 moderately associates wtp53 in cancer cells (Fig. 1b, c, i). Thus, we wondered if TRIM71 plays a role in the wtp53-expressed ovarian cancer cell line OVCAR433 by inhibiting wtp53 activity. Surprisingly, knockdown of TRIM71 barely affected wtp53 expression (Fig. S7a), proliferation (Fig. S7b) or invasion of the cells (Fig. S7c, d). Therefore, our results not only suggest that TRIM71 might have little impact on wtp53 function in ovarian cancer, probably owing to its lower binding affinity to the latter, but again support that the tumor suppressive function of TRIM71 is through inactivation of mtp53s in ovarian cancer.

### TRIM71 suppresses growth of ovarian cancer through inactivation of mutant p53 in vivo

**Fig. 6:**
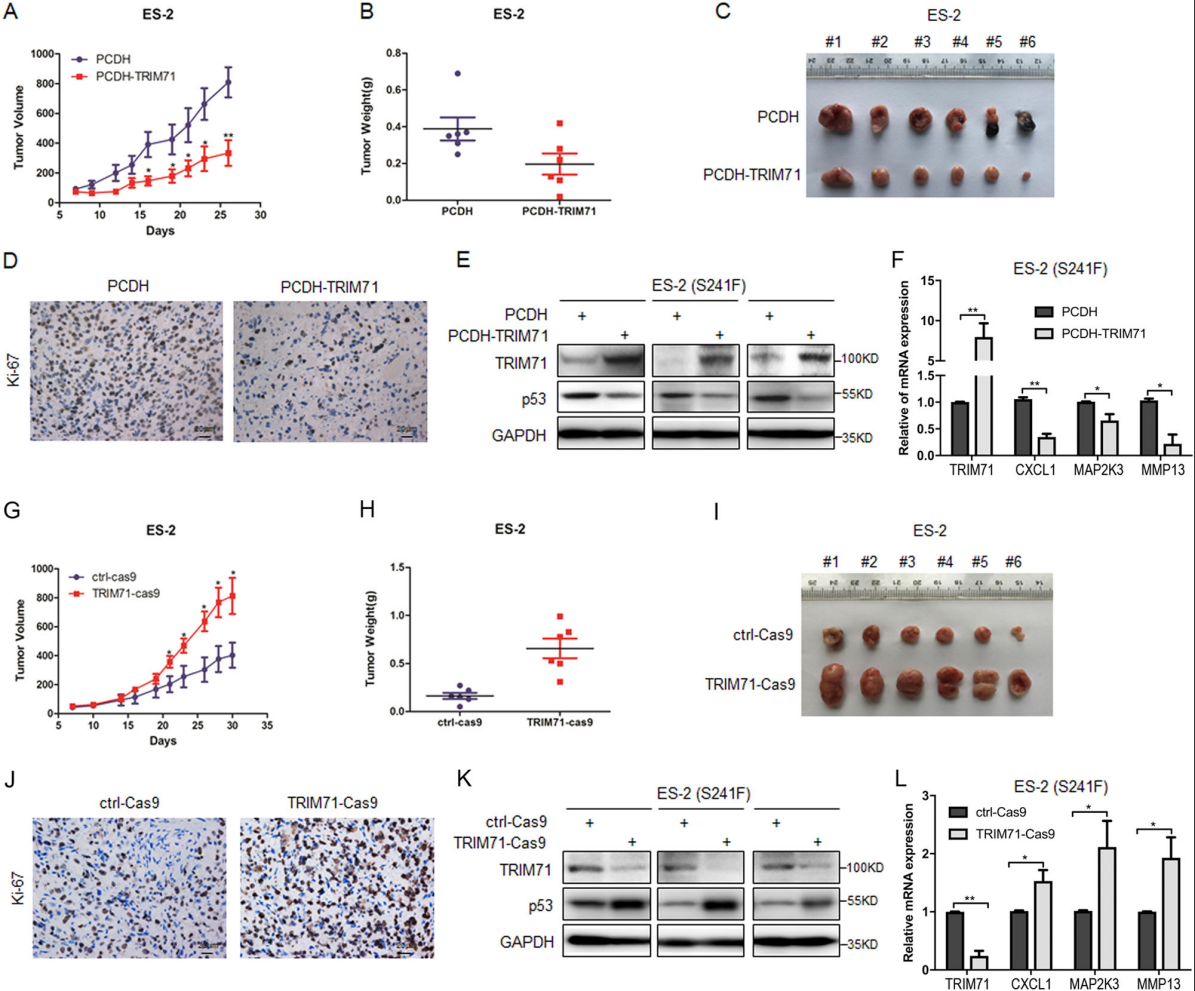
TRIM71 suppresses tumor growth in vivo. **a** ES-2 cells stably overexpressing TRIM71 reveal less tumor volume in average, monitored during growth of the xenograft tumors. **b**, **c** The dissected tumors show that stable overexpression of TRIM71 reduces tumor weight and mass. Data are represented as mean ± SD, *n* = 6. **d** Representative images of Ki-67 staining of the xenograft tumors indicate that TRIM71 overexpression suppresses cell proliferation in vivo. **e** TRIM71 inhibits mtp53 protein expression in vivo by IB assays using antibodies as indicated. **f** The mRNA levels of TRIM71 and mtp53 target genes, CXCL1, MAP2K3, and MMP13, were detected by RT-qPCR analysis (mean ± SD) of three pairs of xenograft tumors. **g** CRISPR/Cas9-mediated depletion of TRIM71 in ES-2 cells elevates tumor volume in average, monitored during growth of the xenograft tumors. **h**, **i** The dissected tumors show that depletion of TRIM71 increases tumor weight and mass. Data are represented as mean ± SD, *n* = 6. **j** Representative images of Ki-67 staining of the xenograft tumors indicate that TRIM71 depletion enhances cell proliferation in vivo. **k** TRIM71 depletion bolsters mtp53 protein expression in vivo by IB assays using antibodies as indicated. **l** The mRNA levels of TRIM71 and mtp53 target genes, CXCL1, MAP2K3 and MMP13, were detected by RT-qPCR analysis (mean ± SD) of three pairs of xenograft tumors. *p < 0.05, **p < 0.01 by two tailed *t* test

To further expand the biological significance of our findings, we established ovarian tumor xenograft models by subcutaneously inoculating ES-2 cells with stably overexpressed or depleted TRIM71 as described above into nude mice. In agreement with the cell-based results, overexpression of TRIM71 significantly repressed tumor growth in vivo compared with the control group as indicated by the tumor volumes (Fig. 6a). Accordingly, the tumor weight and mass were significantly reduced upon TRIM71 overexpression (Fig. 6b, c). In addition, the TRIM71-overexpressed tumor samples exhibited strikingly reduced Ki-67 signals, indicating retarded proliferation of these tumor cells (Fig. 6d). Furthermore, the IB and RT-qPCR analyses of the tumors revealed that overexpression of TRIM71 is correlated with declined expression of mtp53 as well as its target genes in vivo (Fig. 6e, f). Conversely, TRIM71 depletion remarkably promoted tumor growth (Fig. 6g), consequently leading to increased tumor weight and mass compared with the control group (Fig. 6h, i). The assessment of Ki-67 expression revealed that tumors with depleted TRIM71 manifests enhanced cell proliferation (Fig. 6j), which could be due to the upregulation of mtp53 and its target genes (Fig. 6k, l). Altogether, our results substantiate the concept that TRIM71 suppresses ovarian malignancy both in vitro and in vivo through the inactivation of mtp53.

### Clinical significance of the TRIM71–mtp53 axis

To translate the above results into clinical significance, we examined the expression of TRIM71 in normal and cancerous cell lines as well as various cancer tissues. First, we found that TRIM71 is highly expressed in ovarian epithelial cells HOSE compared with cancer cells (Fig. S8a). Also, TRIM71 was underexpressed in multiple human cancers, such as cervix, esophagus, lung, and kidney carcinomas, in the Oncomine database (www.oncomine.org) (Fig. S8b–e). We then performed the Kaplan–Meier survival analysis (kmplot.com) and found that the higher level of TRIM71 is associated with favorable prognosis of cervix, head and neck, kidney, pancreas, thymus, and thyroid carcinomas (Fig. S8f–k). These observations suggest that TRIM71 may act as a tumor suppressor through both mtp53-dependent and independent manners in the context of different cancers, as cervix, thymus and thyroid carcinomas usually have less *TP53* mutations.

**Fig. 7:**
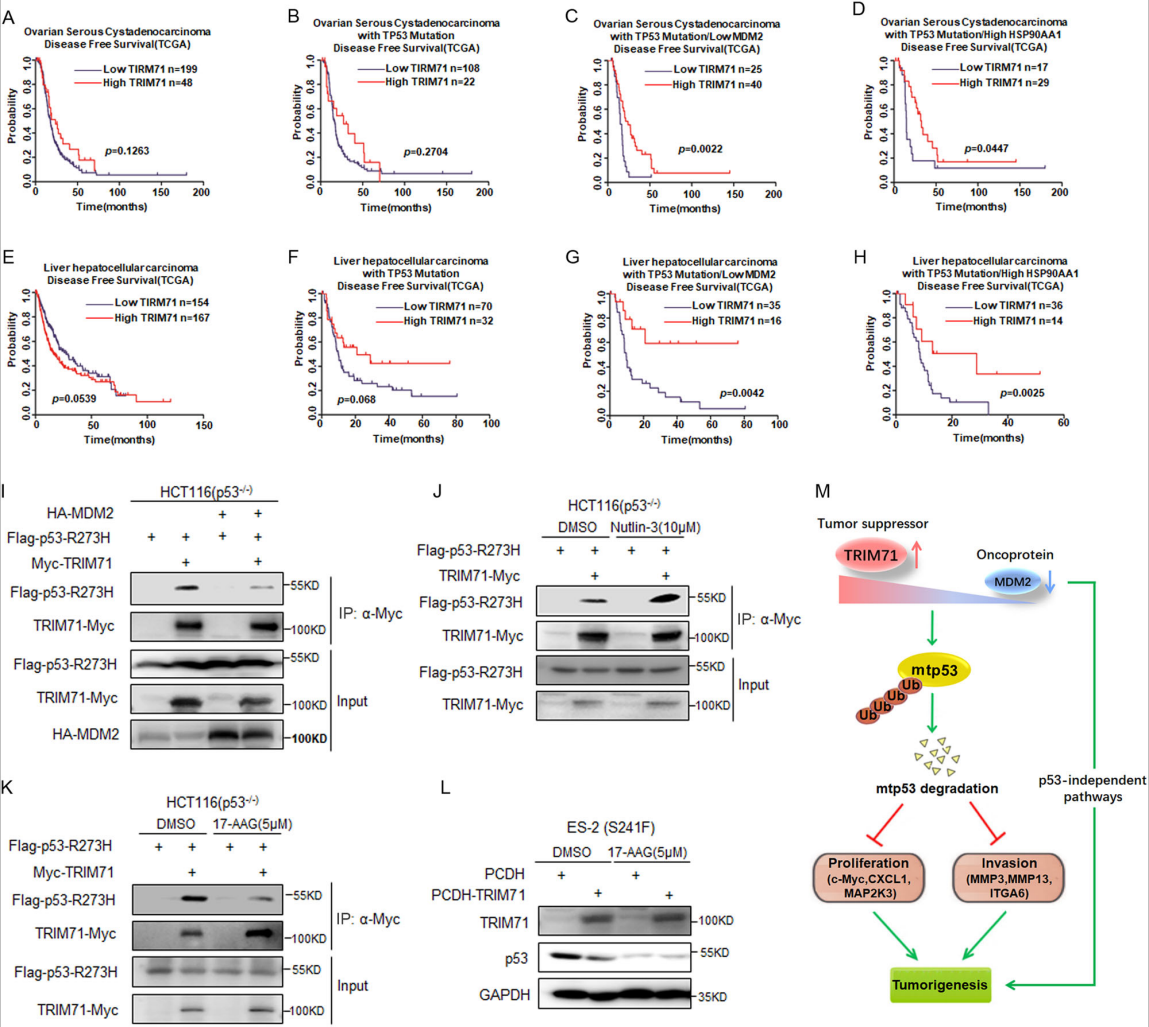
TRIM71 is associated with favorable prognosis in cancer. **a** The Kaplan–Meier analysis reveals that high expression of TRIM71 moderately improves disease-free survival of ovarian cancer patients. **b** High expression of TRIM71 moderately improves disease-free survival of the *TP53*-mutated ovarian cancer patients. **c** High expression of TRIM71 significantly improves disease-free survival of the ovarian cancer patients sustaining *TP53* mutations and low level of MDM2. **d** High expression of TRIM71 significantly improves disease-free survival of the ovarian cancer patients sustaining *TP53* mutations and high level of HSP90. **e**, **f** The Kaplan–Meier analyses reveal that high expression of TRIM71 moderately improves disease-free survival of the *TP53*-mutated liver hepatocellular cancer patients **f**, but not the overall liver hepatocellular cancer patients **e**. **g** High expression of TRIM71 significantly improves disease-free survival of the liver hepatocellular cancer patients sustaining *TP53* mutations and low level of MDM2. **h** High expression of TRIM71 significantly improves disease-free survival of the hepatocellular cancer patients sustaining *TP53* mutations and high level of HSP90. **i** Overexpression of MDM2 attenuates the interaction between TRIM71 and mtp53-R273H. HCT116^p53−/−^ cells were transfected with combinations of plasmids encoding HA-MDM2, Myc-TRIM71, and Flag-p53-R273H followed by co-IP-IB assays using antibodies as indicated. MG132 (20 μm) was supplemented for 6 h prior to cell harvest. **j**, **k** Nutlin-3 treatment enhances **j**, whereas 17-AAG treatment suppresses **k**, the interaction between TRIM71 and mtp53-R273H. HCT116^p53−/−^ cells were transfected with plasmids encoding Myc-TRIM71 and Flag-p53-R273H followed by co-IP-IB assays using antibodies as indicated. Nutlin-3 (10 μm) or 17-AAG (5 μm) was supplemented for 24 h, and MG132 (20 μm) was supplemented for 6 h prior to cell harvest. **l** TRIM71 is unable to induce mtp53 degradation in the presence of 17-AAG. ES-2 cells infected with control or TRIM71-expressing lentiviruses were treated with or without 17-AAG (5 μm) for 24 h followed by the IB analysis using antibodies as indicated. **m** The working model for TRIM71’s tumor-suppressive function by inducing mtp53 degradation and consequently preventing tumor growth and metastasis

Next, we investigated the clinical relevance of TRIM71 in ovarian cancer. To our surprise, the preliminary analysis showed that TRIM71 exhibits no significant prognostic value in the overall ovarian cancer patients or the *TP53*-mutated ovarian cancer patients, although high level of TRIM71 is moderately associated with survival of these patients (Fig. 7a, b). It is known that MDM2 is the master E3-ubiquitin ligase toward both wt and mtp53s by interacting with the TA domain^27,32^ that is also required for TRIM71 interaction, and that MDM2 is encoded by an oncogene often amplified or overexpressed in cancer^33^. We speculated that the E3 activity of MDM2 might be dominant in the degradation of mtp53, leading to the crippled TRIM71–mtp53 axis and thus impaired prognostic significance of TRIM71. To minimize the impact of MDM2, we selectively studied the *TP53*-mutated ovarian cancer patients sustaining underexpressed MDM2. Remarkably, in this cohort of patients, high level of TRIM71 was significantly associated with improved patient survival (Fig. 7c). To confirm this observation, we evaluated the survival probability in the cohort of the HSP90-highly expressed, *TP53*-mutated ovarian cancer patients, because HSP90 was shown to considerably dampen MDM2-E3 activity in cancer^34,35^. Again, high expression of TRIM71 was significantly associated with favorable prognosis of these patients (Fig. 7d). These results also imply that TRIM71 and MDM2 could be combined prognostic biomarkers in the *TP53*-mutated ovarian cancers. Consistently, we also validated the clinical significance of the TRIM71–mtp53 cascade in liver cancer. As shown in Fig. 7e–h, high expression of TRIM71 was selectively and significantly associated with improved prognosis in the MDM2-depressed, *TP53*-mutated liver cancer patients (Fig. 7g, h). Collectively, these observations suggest that high level of MDM2 undermines, whereas inhibition of MDM2 enhances, the TRIM71–mtp53 axis in cancer. To dissect the molecular basis, we performed a series of co-IP assays and found that ectopically overexpressed MDM2 drastically attenuates the binding of TRIM71 to mtp53 (Fig. 7i). Conversely, this binding was significantly augmented when cells were treated with the MDM2 antagonist Nutlin-3 (Fig. 7j) that selectively disrupts MDM2 interactions with wt and mtp53^36,37^. In addition, we also showed that the HSP90 inhibitor 17-AAG, which prompts mtp53 degradation by MDM2 impairs the TRIM71–mtp53 interaction (Fig. 7k) and, as thus, prevents TRIM71-induced mtp53 degradation (Fig. 7l). Taken together, these results demonstrate the interplay between MDM2 and TRIM71 in regulating mtp53 activity, and further emphasize the biological and clinical significance of the latter in primary cancer development and prognosis.

## Discussion

A thorough elucidation of the mechanisms underlying control of mtp53 activity would provide useful information for future development of anticancer therapies. In this study, we identified the E3-ubiquitin ligase TRIM71 as a mtp53-interacting protein in ovarian cancer. Interestingly, TRIM71 bound to all the p53 mutants examined (Fig. 1), via the mtp53-TA domain and the TRIM71-NHL domain (Fig. 2). Of note, our results together with others reveal that both TRIM71 and MDM2 bind to the same region of mtp53s^27,32^, suggesting that they could play an overlapping role in regulating mtp53 (Fig. 7a–l), which will be further discussed below. Also interestingly, we identified four lysines of the ubiquitin, including K11, K27, K29, and K63, critical for TRIM71-mediated mtp53 poly-ubiquitination, as mutating each of them impaired mtp53 ubiquitination by this E3 ligase (Fig. 3l, m). Although ubiquitin chains linked by these lysines have been found to promote proteolysis of the substrates^29^, which is consistent with our result showing that TRIM71 can degrade mtp53 (Fig. 3), the K29-, K27-, and K63-linked chains have also been shown to modulate protein interactions, activity, or cellular localization of target substrates^29^. Thus, it remains to be studied if TRIM71 might control mtp53 activity through proteolysis-independent mechanisms. Collectively, these findings at least demonstrate for the first time that the E3-ubiquitin ligase TRIM71 can directly bind to and target mtp53s for proteasomal degradation.

In line with the above results, our functional analyses also revealed that TRIM71 represses the expression of mtp53 target genes responsible for the implementation of different mtp53s’ GOFs, such as cell survival, proliferation, and metastasis (Fig. 4a, b). The downregulation of mtp53 target genes by TRIM71 was also verified in primary cancers harboring mtp53s, as a significant inverse correlation between the expression of TRIM71 and mtp53 target genes was found in ovarian cancer, but not in the prostate cancers with less *TP53* mutations (Fig. 4c–j, S1–S3). Consistent with our findings, TRIM71 has also been shown to have a tumor suppressive role in lung and colon cancer cells by mediating degradation of LIN28B and consequently upregulating the tumor suppressive microRNA let-7^38,39^. But, it has also been reported that TRIM71 might act as an oncoprotein to activate the NF-κB signaling pathway by degrading IκB-α, or repress the Argonautes-microRNA pathway, leading to development of lung and liver carcinomas^40,41^. These divergent findings may reflect the complexity and heterogeneity of cancers. However, regardless of these differences, our findings as described here strongly support the concept that TRIM71 can at least act as a tumor suppressor to repress the growth and migration of ovarian cancer cells (Fig. 5 and 6, and S6) by suppressing mtp53 activity and, consequently, the survival- and metastasis-associated target genes (Fig. 4). Surprisingly, knockdown of TRIM71 had no effect on the growth and migration of wtp53-expressed ovarian cancer cells (Fig. S7), indicating that TRIM71 might only regulate the level and activity of mt, but not wt, p53 in ovarian cancer cells. Moreover, in line with a previous report showing that TRIM71 is downregulated in a variety of cancers including ovarian carcinoma^39^, we also found that TRIM71 is underexpressed in multiple ovarian cancer cell lines (Fig. S8a) and associated with improved prognosis of ovarian malignancy (Fig. 7c, d). Altogether, these results convincingly demonstrate that TRIM71 functions as a tumor suppressor by globally prohibiting the expression of mtp53 target genes in *TP53*-mutated ovarian cancers.

Significantly, our bioinformatics analysis of primary ovarian cancer data via online available information uncovers that TRIM71 could be a prognostic marker for ovarian cancer. Overexpression of TRIM71 is associated with favorable prognosis through perturbation of the mtp53 signaling pathways particularly when MDM2 is underexpressed or inactivated (Fig. 7a–h), which is probably owing to the fact that high level of MDM2 may prevent the TRIM71–mtp53 interaction (Fig. 7i–l). These analyses together with our experimental results as shown above also suggest the TRIM71–mtp53 axis might be targetable for future development of therapy against this type of cancer. Targeting mtp53 for degradation has been attested to be an efficient strategy for treatment of cancer^2^. For instance, the HSP90 inhibitors, such as Geldanamycin and Ganetespib, were found to destabilize mtp53s by eliciting the E3 ligase activity of MDM2^34,35^. However, the activation of MDM2 by these potential anticancer compounds may trigger the p53-independent oncogenic activity of MDM2^42,43^. Another hidden problem for targeting MDM2 as an anticancer therapy is that inactivation of MDM2 could otherwise result in the increase of mtp53 expression if cancers harbor hotspot mtp53s. Thus, co-targeting the TRIM71- and MDM2-mtp53 pathways might be a better strategy for the development of potential therapies for this type of cancers (Fig. 7m).

In conclusion, our results as shown here demonstrate that the E3-ubiquitin ligase TRIM71 binds to mtp53s, and mediates their ubiquitination and proteasomal degradation, leading to inhibition of the mtp53 downstream signaling pathways (Fig. 7m). Consistently, overexpression of TRIM71 can suppress, whereas ablation of TRIM71 can boost, the growth and metastasis of ovarian cancer cells in vitro and the growth of ovarian cancer in vivo by influencing mtp53 activity. Significantly, TRIM71 overexpression in tumors is well correlated with downregulation of mtp53 target genes, which further predicts favorable prognosis of ovarian cancer patients. Our findings also suggest the possibility of targeting the TRIM71–mtp53 axis as a potential strategy for future development of anticancer therapies for mtp53-expressed ovarian carcinomas and perhaps other types of cancers that harbor mtp53s.

## Supplementary information

Supplementary Figures

Supplementary figure legend
