# Supplementary material for: Ubiquitin ligase TRIM71 suppresses ovarian tumorigenesis by degrading mutant p53
Source: Cell Death Dis. 2019 Sep 30;10(10):737. doi: 10.1038/s41419-019-1977-3 (PMC6769007; doi:10.1038/s41419-019-1977-3)

Figure S1 (related to Figure 4)

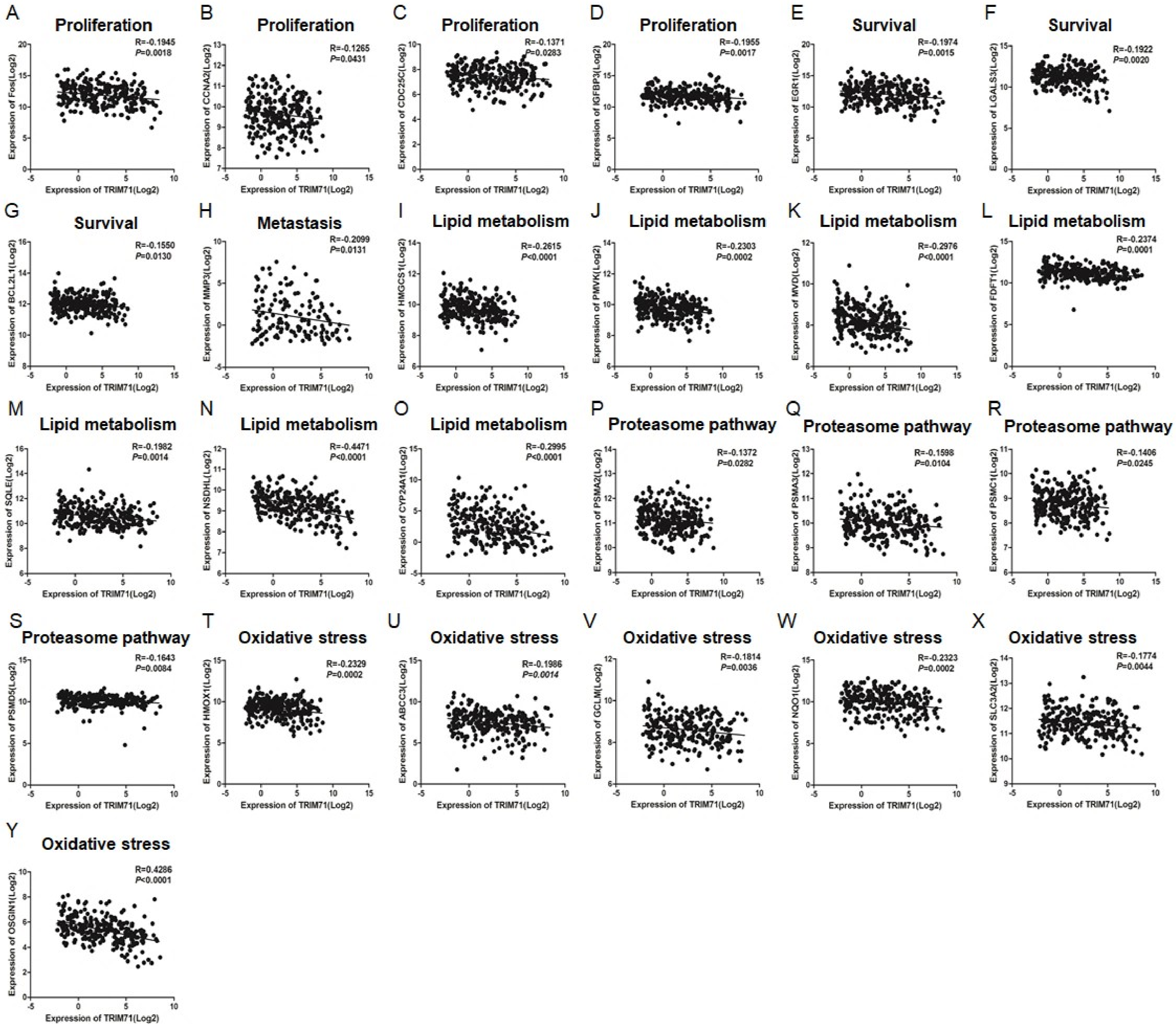

Figure S2 (compared with Figure 4)

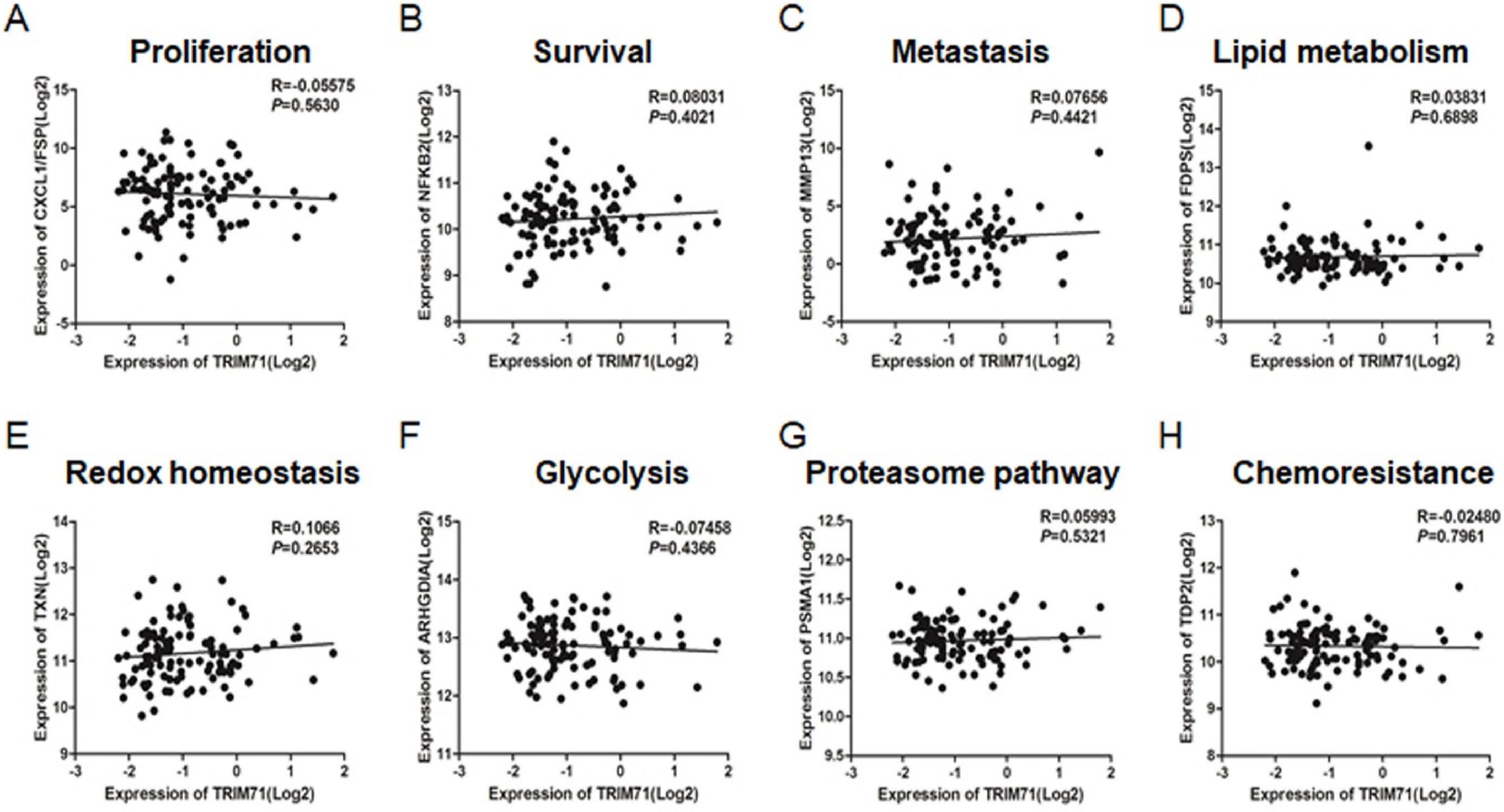

Figure S3 (compared with Figure S1)

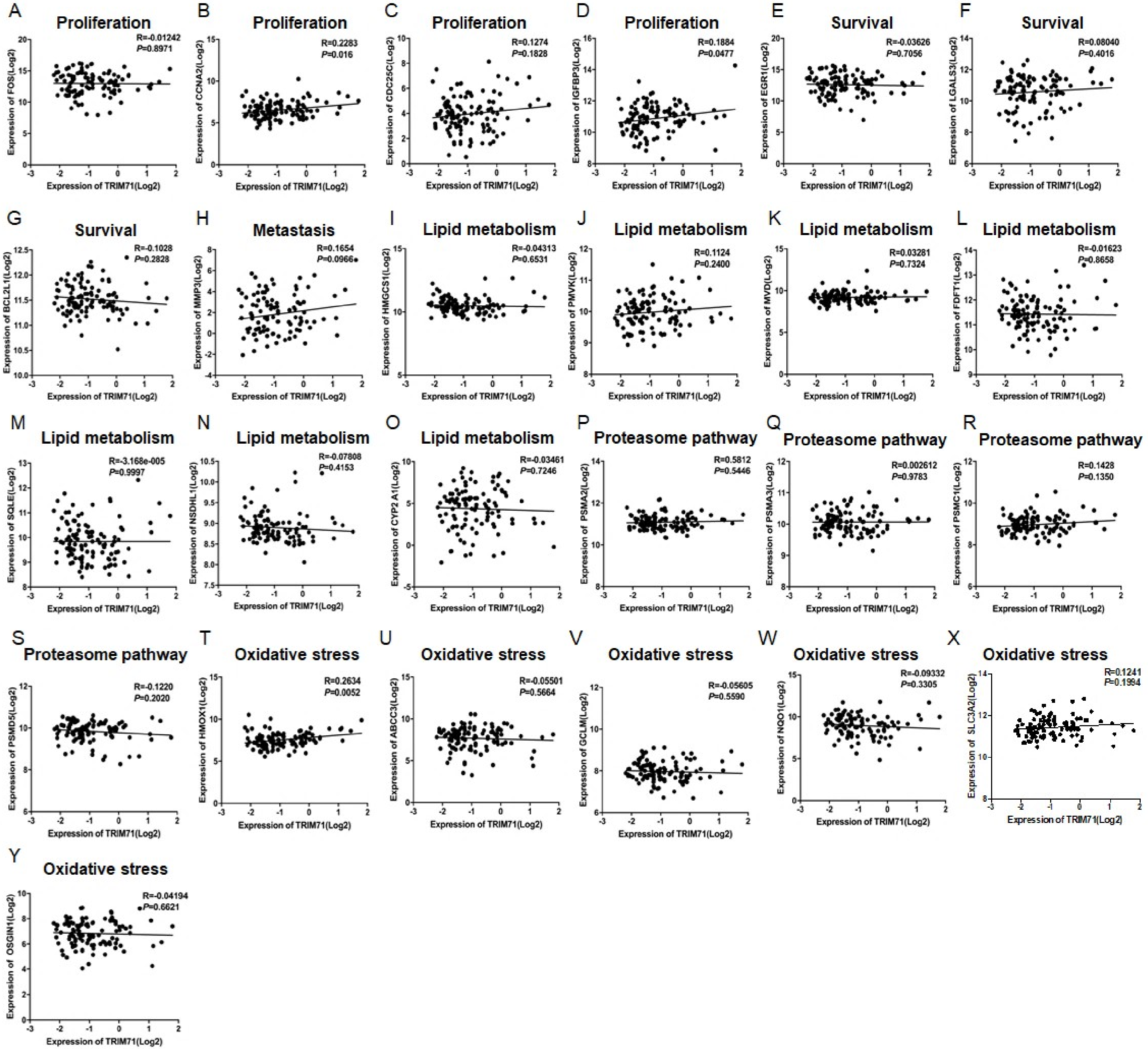

Figure S4

A

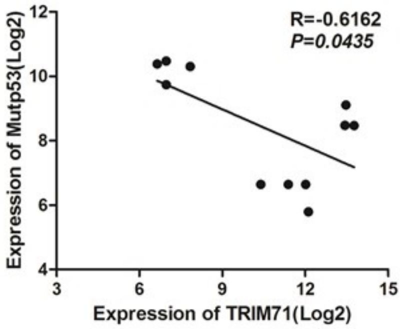

B

TP53-exon sequencing of 11 ovarian cancer samples with missense mutations

Sample NO.	Position	TP53 Mutation	Amino acids	Flanking Sequences
1	TP53-Exon 5	CAT-GAT	H179D	GTGAGGCGCTGCCCCCAC(C/CG)ATGAGCGCTGCTCAGATAGCGA
2	TP53-Exon 8	CGT-CCT	R273P	ACGGAACAGCTTTGAGGTGC(G/GC)TGTTTGTGCCTGTCCTGGGA
3	TP53-Exon 7	CGG-TGG	R248W	CCTGCATGGGCGGCATGAAC(C/CT)GGAGGCCCATCCTCACCATC
4	TP53-Exon 7	TCC-TTC	S241F	CAACTACATGTGTAACAGTT(C/T)CTGCATGGGCGGCATGAACC
5	TP53-Exon 8	CGT-TGT	R273C	GACGGAACAGCTTTGAGGTG(C/CT)GTGTTTGTGCCTGTCCTGGG
6	TP53-Exon 7	GGC-AGC	G245S	GTAACAGTTCCTGCATGGGC(G/GA)GCATGAACCGGAGGCCCATC
7	TP53-Exon 6	TAT-TGT	Y220C	ACATAGTGTGGTGGTGCCCT(A/AG)TGAGCCGCCTGAGGTCTGGT
8	TP53-Exon 7	ATG-GTG	M246V	ACAGTTCCTGCATGGGCGGC(A/G)TGAACCGGAGGCCCATCCTC
9	TP53-Exon 6	TAT-TGT	Y205C	AGGAAATTTGCGTGTGGAGT(A/AG)TTTGGATGACAGAAACACTT
10	TP53-Exon 5	CAT-CAG	H179Q	GTGAGGCGCTGCCCCCACCA(T/TG)GAGCGCTGCTCAGATAGCGA
11	TP53-Exon 6	ATC-AAC	I195N	GGCCCCTCCTCAGCATCTTA(T/TA)CCGAGTGGAAGGAAATTTGC

C

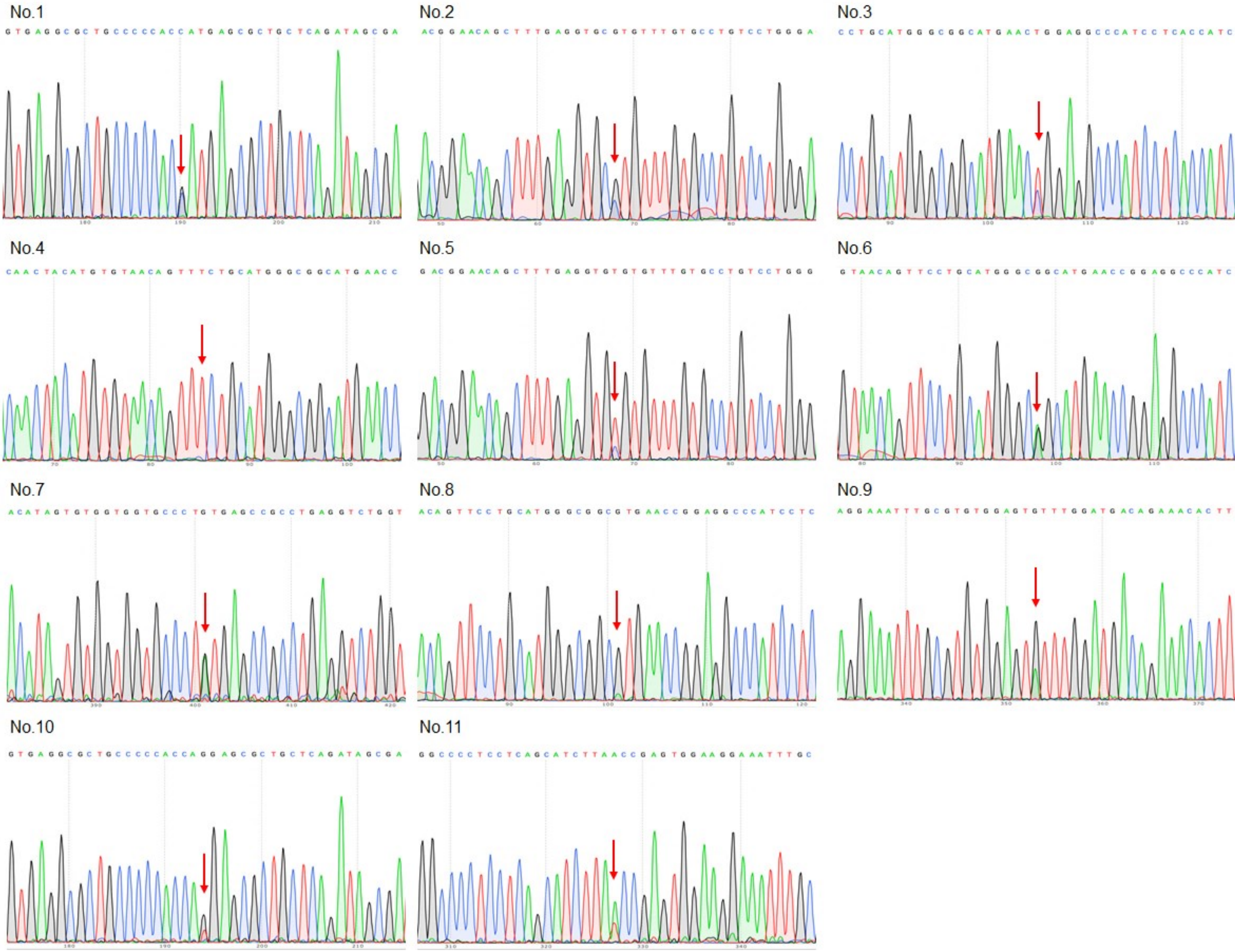

Figure S5 (related to Figure 5)

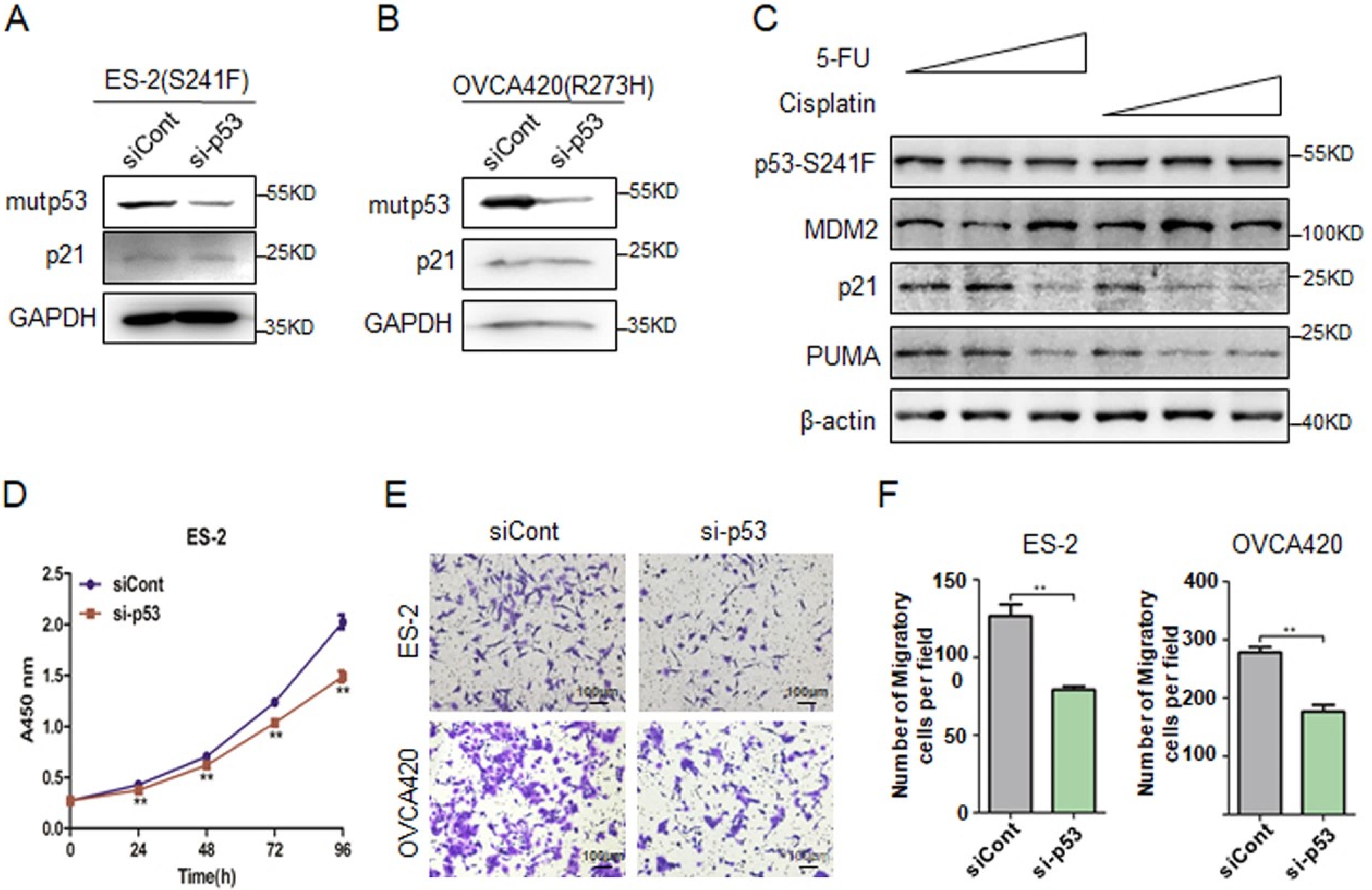

Figure S6 (related to Figure 5)

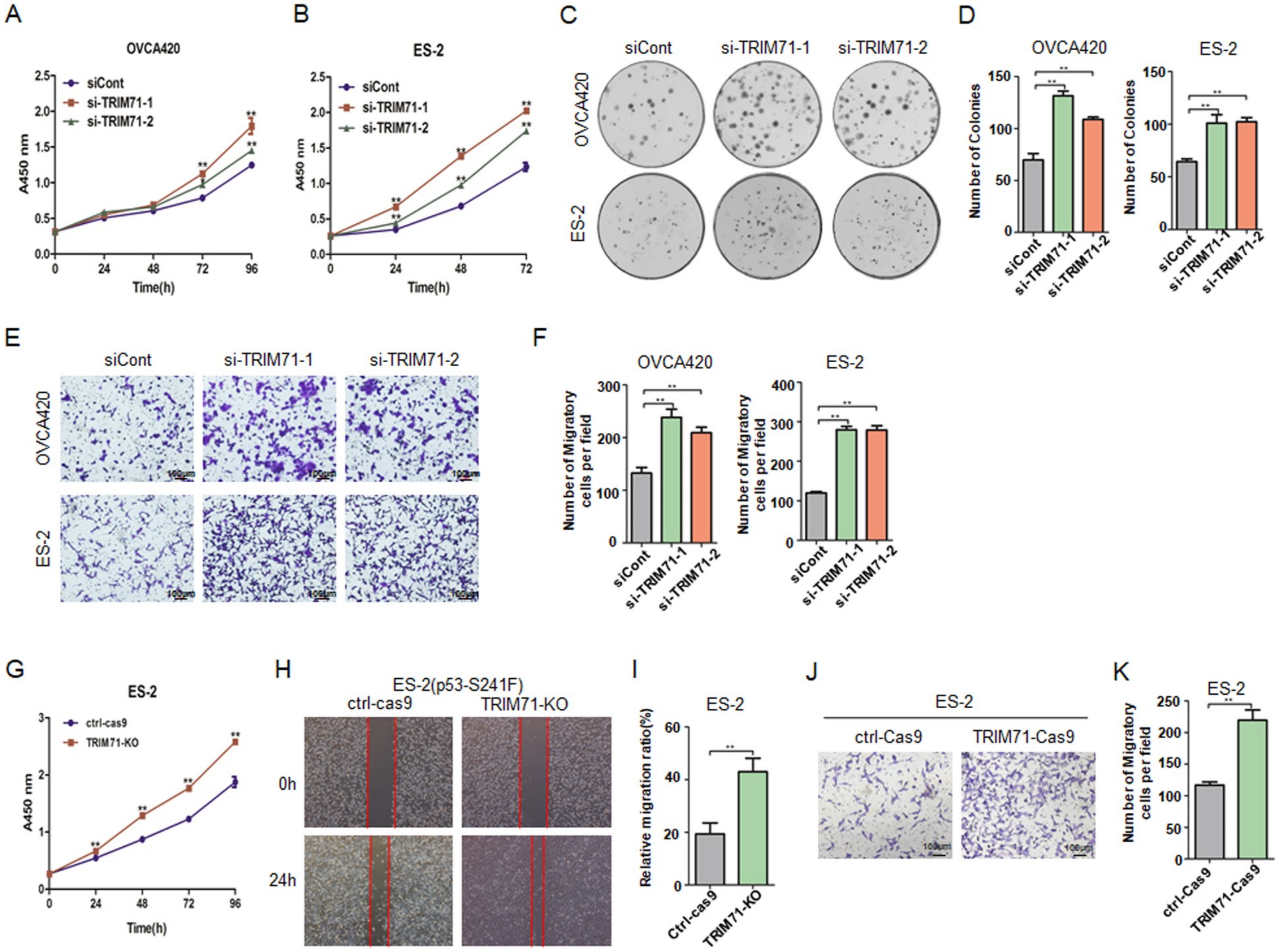

Figure S7

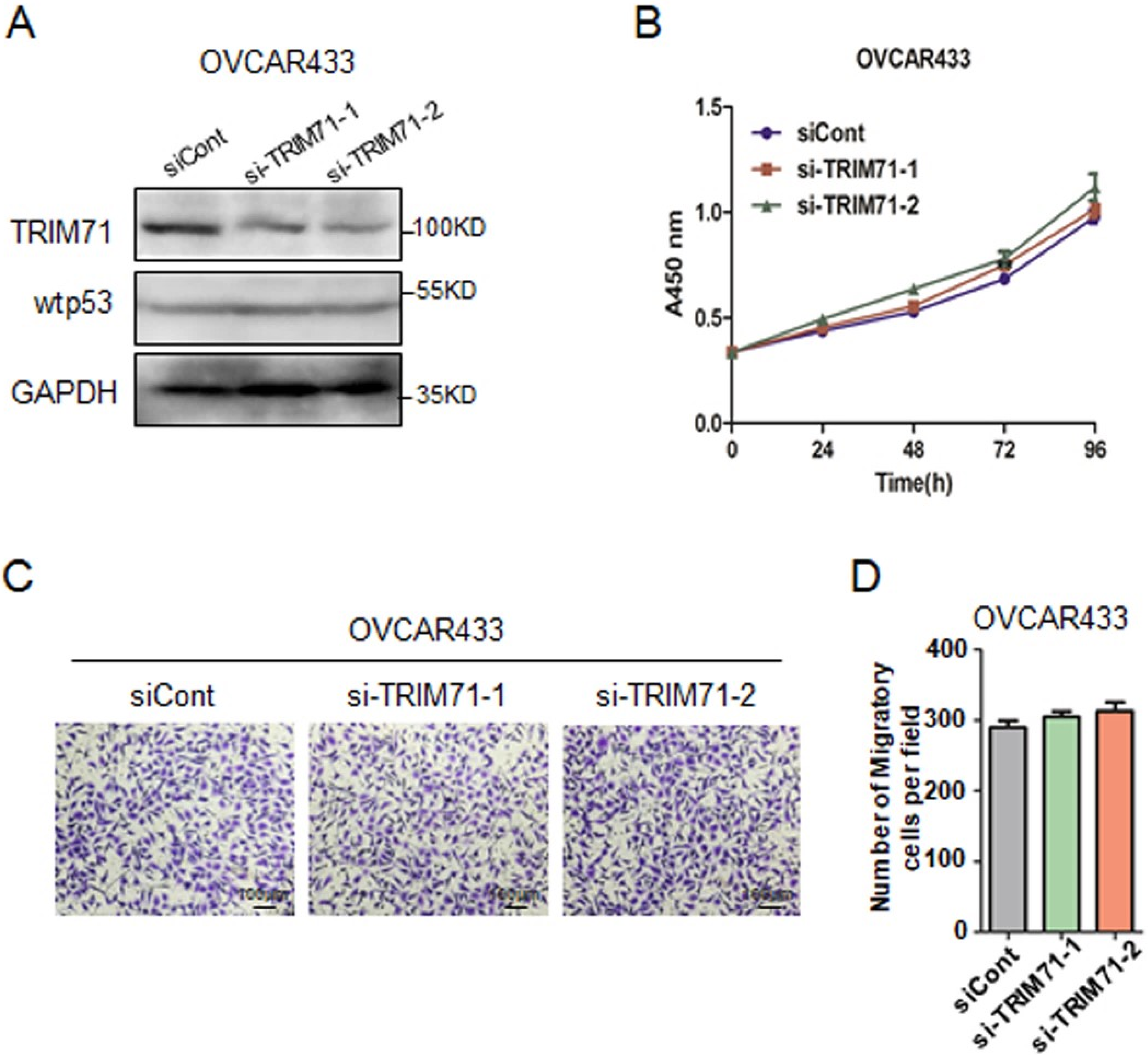
A
OVCAR433
siCont
si-TRIM71-1
si-TRIM71-2
TRIM71
wtp53
GAPDH
100KD
55KD
35KD
B
OVCAR433
siCont
si-TRIM71-1
si-TRIM71-2
A450 nm
Time(h)
0.0
0.5
1.0
1.5
0
24
48
72
96
C
OVCAR433
siCont
si-TRIM71-1
si-TRIM71-2
D
OVCAR433
Number of Migratory cells per field
0
100
200
300
400
siCont
si-TRIM71-1
si-TRIM71-2

Figure S8 (related to Figure7)

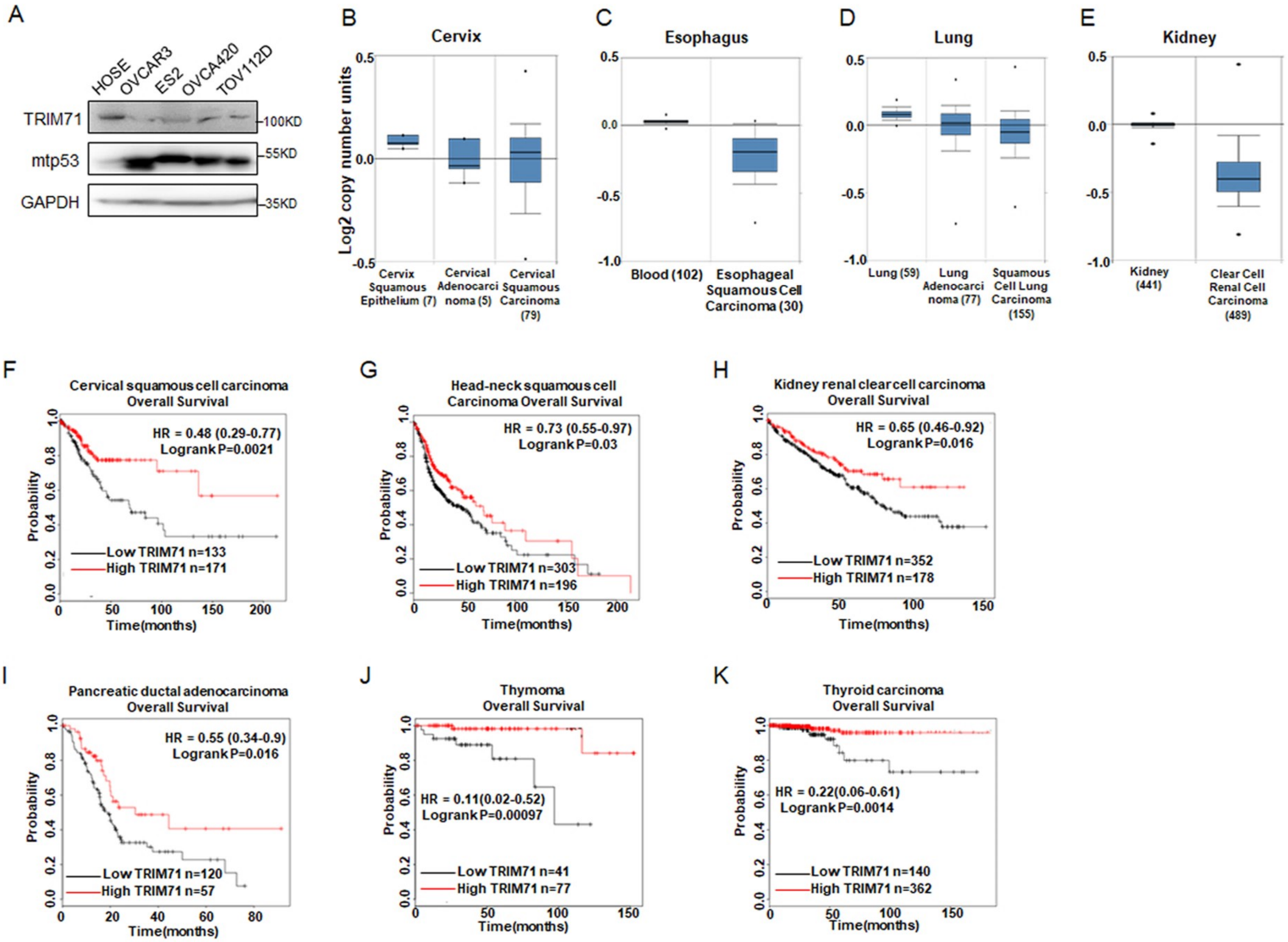

Supplement: Supplementary file 1 — Supplementary Figures [file 41419_2019_1977_MOESM1_ESM.pdf]
