# Supplementary material for: Ubiquitin ligase TRIM71 suppresses ovarian tumorigenesis by degrading mutant p53
Source: Cell Death Dis. 2019 Sep 30;10(10):737. doi: 10.1038/s41419-019-1977-3 (PMC6769007; doi:10.1038/s41419-019-1977-3)
Supplement: Supplementary file 2 — Supplementary figure legend [file 41419_2019_1977_MOESM2_ESM.docx]

Supplementary Information for

Ubiquitin ligase TRIM71 suppresses ovarian tumorigenesis by degrading mutant p53

Yajie Chen, Qian Hao, Jieqiong Wang, Jiajia Li, Canhua Huang, Yu Zhang, Xiaohua Wu, Hua Lu^*^, Xiang Zhou^*^

^*^Corresponding Authors:

Xiang Zhou, Fudan University Shanghai Cancer Center and Institutes of Biomedical Sciences, Fudan University, Shanghai 200032, P. R. China. Email: [xiangzhou@fudan.edu.cn](mailto:xiangzhou@fudan.edu.cn)

Hua Lu, Department of Biochemistry & Molecular Biology and Tulane Cancer Center, Tulane University School of Medicine, New Orleans, LA 70112, USA. Email: [hlu2@tulane.edu](mailto:hlu2@tulane.edu)

**This PDF file includes:**

Supplementary Figure legends

Supplementary Figures S1 to S8

**Supplementary Figure legends**

**Supplementary Figure S1. TRIM71 expression is negatively correlated with the mtp53 signaling pathways in ovarian cancer.** (A-Y) The inverse correlation between the expression of TRIM71 and the mtp53 target genes, involved in proliferation (A-D), survival (E-G), metastasis (H), lipid metabolism (I-O), proteasome pathway (P-S), and oxidative stress (T-Y) in ovarian cancer (TCGA database).

**Supplementary Figure S2. No significant correlation between the expression of TRIM71 and the mtp53 target genes is found in prostate cancer with less frequency of *TP53* mutation.** Eight mtp53 target genes shown in Figure 4C-4J were analyzed in 498 prostate cancer tumors from the TCGA database.

**Supplementary Figure S3. No significant negative correlation between the expression of TRIM71 and 25 mtp53 target genes is found in prostate cancer.** (A-D) are proliferation-associated genes, (E-G) are survival-associated genes, (H) is a metastasis-associated gene, (I-O) are lipid metabolism-associated genes, (P-S) are proteasome pathway-associated genes, and (T-Y) are oxidative stress-associated genes.

**Supplementary Figure S4. The expression of mtp53 is negatively associated with TRIM71 in ovarian cancer samples.** (A) Pearson’s correlation curves reveals the negative relationship between the protein expression of TRIM71 and mtp53, n = 11. The ovarian cancer samples were subjected to TP53-exon sequencing, and those with single mutation were subjected to the IB assay. (B, C) A list of the TP53 missense mutations of 11 ovarian cancer samples. The red arrows indicate the mutated bases (C).

**Supplementary Figure S5. p53-S241F and R273H are oncogenic proteins in ovarian cancer.** (A, B) Knockdown of mtp53s does not regulate p21 expression in ES-2 and OVCA420 cells. (C) p53-S241F in ES-2 cells is not responsive to DNA damage stress triggered by 5-FU or Cisplatin. (D) Knockdown of p53-S241F inhibits proliferation of ES-2 cells. (E, F) Knockdown of p53-S241F or R273H inhibits invasion of ES-2 or OVCA420 cells. The representative images and quantification analysis are shown in (E) and (F), respectively.

**Supplementary Figure S6. Depletion of TRIM71 inhibits ovarian cancer cell growth and metastasis**. (A, B) Knockdown of TRIM71 by two independent siRNAs prompts proliferation of OVCA420 and ES-2 cells by the CCK-8 assays. (C, D) Knockdown of TRIM71 enhances the colony-forming ability of OVCA420 and ES-2 cells. The representative images and quantification analysis are shown in (C) and (D), respectively. (E, F) Knockdown of TRIM71 promotes invasion of OVCA420 and ES-2 cells by the transwell assays. The representative images and quantification analysis are shown in (E) and (F), respectively. (G) Depletion of TRIM71 in ES-2 cells by CRISPR/Cas9 prompts cell proliferation. (H, I) Depletion of TRIM71 in ES-2 cells by CRISPR/Cas9 promotes wound-healing migration. The representative images and quantification analysis are shown in (H) and (I), respectively. (J, K) Depletion of TRIM71 in ES-2 cells by CRISPR/Cas9 promotes cell invasion. The representative images and quantification analysis are shown in (J) and (K), respectively.

**Supplementary Figure S7. TRIM71 is not required for proliferation or invasion of OVCAR433 cells harboring wtp53.** (A) Knockdown of TRIM71 by two independent siRNAs does not affect wtp53 protein levels in OVCA433 cells. (B) Knockdown of TRIM71 does not affect OVCAR433 cell proliferation. (C, D) Knockdown of TRIM71 does not affect OVCAR433 cell invasion. The representative images and quantification analysis are shown in (C) and (D), respectively.

**Supplementary Figure S8. The expression of TRIM71 is reduced in carcinomas over normal tissues, which is associated with poor prognosis.** (A) The expression of TRIM71 is lower in several ovarian carcinoma cell lines, OVCAR3, ES-2, OVCA420, and TOV112D, than that in the ovarian epithelial cell line HOSE. (B-E) The TRIM71 copy number is reduced in carcinomas compared to the normal tissues. The data were collected from the Oncomine database. (F-K) The Kaplan–Meier survival analyses indicate that high expression of TRIM71 predicts improved patient survival in cervical squamous cell carcinoma (F), head and neck squamous cell carcinoma (G), kidney renal clear cell carcinoma (H), pancreatic ductal adenocarcinoma (I), thymoma (J), and thyroid carcinoma (K).
